# Phosphor‐Sensitized Ternary Organic Solar Cells Achieving 18.6% PCE via Ir(III)‐Mediated Triplet Energy Transfer and Non‐Radiative Recombination Suppression

**DOI:** 10.1002/advs.78199

**Published:** 2026-10-08

**Authors:** Priyanka Yadav, Guohong Liu, Hyerin Kim, Songhyun Kim, Minsung Kim, Yeongbin Ma, Jisan Lim, Young Yong Kim, Taehyung Jang, Raja Kumaresan, Jinhwan Yoon, Sung‐Ho Jin

**Affiliations:** ^1^ Department of Chemistry Education Graduate Department of Chemical Materials Institute for Plastic Information and Energy Materials Sustainable Utilization of Photovoltaic Energy Research Center (ERC) Pusan National University Busan Republic of Korea; ^2^ Beamline Division Pohang Accelerator Laboratory Pohang University of Science and Technology Pohang‐si Republic of Korea; ^3^ Energy Spectroscopy Laboratory Department of Chemistry Gwangju Institute of Science and Technology (GIST) Gwangju Republic of Korea; ^4^ Department of Chemical Engineering Center for Innovative Chemical Processes Institute of Engineering University of Seoul Seoul Republic of Korea; ^5^ KR Advanced Electronic Materials Co. Ltd Busan Republic of Korea

**Keywords:** Förster resonance energy transfer, host‐guest engineering, Ir‐3 complex, non‐radiative recombination, phosphor‐sensitized organic solar cells, triplet‐triplet energy transfer

## Abstract

Phosphor‐sensitized organic solar cells (PS‐OSCs) enhance exciton utilization and suppress non‐radiative recombination by incorporating triplet‐enabled sensitizers into bulk‐heterojunction blends. Here, we report a green‐emitting Ir‐3 complex as a multifunctional phosphor‐sensitizer in ternary OSCs based on PM6, two newly designed pyrazine‐derived donor polymers, PBDT‐Pyz (N1) and PBDT‐T‐Pyz (N2), and the non‐fullerene acceptor L8‐BO. N1 and N2 exhibit deep HOMO levels (−5.59/−3.63 eV and −5.58/−3.60 eV) and planar backbones for efficient charge transport, while Ir‐3 (E_g_^opt^ = 2.50 eV, HOMO/LUMO −5.50/−3.0 eV) functions simultaneously as an interfacial compatibilizer, luminescent‐cascade promoter, and triplet‐energy mediator. Strong spin‐orbit coupling in Ir(III) enables near‐quantitative intersystem crossing (ISC), populating long‐lived triplet states that undergo rapid triplet‐triplet energy transfer (TTET) to L8‐BO (Ir‐3 *T*_1_ = 2.46 eV → L8‐BO *T*_1_ = 1.85 eV, ΔE_T1_ = 0.61 eV), converting otherwise inaccessible triplet excitons into productive charge generation while suppressing non‐radiative voltage loss. The optimized *o*‐xylene‐processed PM6:N2:L8‐BO device containing 1 wt.% Ir‐3 achieved a champion PCE of 18.6%, with *V*_oc_ of 0.88 V, *J*_sc_ of 27.6 mA cm^−2^, and FF of 76.7%, a 2.6% absolute gain over the binary control. These results establish a combined host‐engineering and phosphor‐sensitization strategy for high‐efficiency, halogen‐free, solvent‐compatible PS‐OSCs.

## Graphical abstract

A synergistic phosphor‐sensitization strategy (PSS) significantly enhances ternary OSCs performance. By integrating an Ir‐3 complex as a triplet energy mediator within a pyrazine‐based (N2) ternary blend (PM6:N2: L8‐BO) doped with 1 wt.% of Ir‐3 complex, and a PCE of 18.6% is achieved through efficient triplet‐triplet energy transfer (TTET), suppressed non‐radiative recombination, and optimized nanoscale morphology.

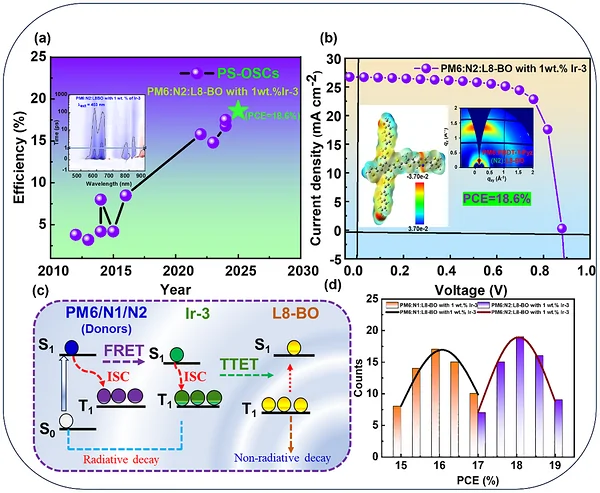

## 1 Introduction

Organic solar cells (OSCs) have emerged as a promising next‐generation energy harvesting technology due to their distinct advantages, which include low manufacturing costs, solution processability, mechanical flexibility, a lightweight nature, and tunable optoelectronic properties [1, 2]. Over the past decade, this field has witnessed remarkable progress, primarily driven by the development of non‐fullerene acceptors (NFAs), particularly the Y‐series derivatives. Combinations of NFAs with wide‐bandgap polymer donors have enhanced the power conversion efficiency (PCE) of single‐junction OSCs beyond the 20% milestone [3, 4]. Among these high‐performance systems, the PM6:L8‐BO blend represents a benchmark, as it exhibits complementary absorption, a favorable bulk‐heterojunction (BHJ) morphology, and significantly less energy loss than traditional fullerene‐based systems [5, 6, 7, 8, 9]. Despite these advancements, state‐of‐the‐art OSCs still face fundamental limitations that prevent performance from reaching the Shockley‐Queisser theoretical limit. Underutilization of the solar spectrum remains a major challenge, as a substantial portion of incident photons, particularly in the low‐energy visible and near‐infrared (NIR) region are either not absorbed or ineffectively converted into electrical current [10]. Furthermore, non‐radiative recombination losses in open‐circuit voltage (*V*_oc_) constitute a critical obstacle, leading to significant *V*_oc_ deficits and overall device efficiency limitations [11, 12]. Addressing these twin challenges to enhance spectral coverage while simultaneously suppressing recombination losses is therefore essential to support the continued advancement of OSC technology. Phosphorescent sensitization using heavy metal complexes offers a means of overcoming these obstacles. In particular, Ir(III) complexes are attractive candidates due to strong spin‐orbit coupling (SOC), which facilitates highly efficient intersystem crossing (ISC) and results in near‐unity phosphorescence quantum yields (PLQYs) and long‐lived triplet excited states [13, 14]. Furthermore, when incorporated into an organic blend, these Ir(III) complexes can function as triplet sensitizers. The operational mechanism involves a multi‐step energy transfer pathway: (i) the sensitizer absorbs photons in spectral regions where the host is transparent, (ii) the excited singlet state undergoes rapid ISC to populate the long‐lived triplet metal‐to‐ligand charge‐transfer (^3^MLCT) state, and (iii) this triplet energy is subsequently transferred to the host donor (D) or acceptor (A) via triplet‐triplet energy transfer (TTET) [15, 16]. This process effectively harvests triplet excitons that are otherwise inaccessible due to the spin‐forbidden nature of direct optical excitation in organic semiconductors. In addition, these triplet excitons, with characteristically longer diffusion lengths, can enhance charge generation efficiency and mitigate geminate recombination [17]. Moreover, the introduction of a phosphor‐sensitizer (PS) with a high‐triplet energy state can create a luminescent cascade and effectively reduce non‐radiative recombination losses and boost attainable *V_oc_* [18, 19]. However, the integration of PS into high‐efficiency systems like PM6:L8‐BO demands precise molecular engineering. An ideal Ir‐3 sensitizer must satisfy several criteria: (1) a triplet energy level (E_T_) sufficiently higher than that of the host material to enable exergonic and irreversible energy transfer, (2) strong absorption in spectral regions complementary to the host blend to maximize photon harvesting, (3) frontier molecular orbital (FMO) levels aligned to prevent the complex from acting as a deep charge trap impeding charge transport, and (4) excellent morphological compatibility to avoid disrupting the finely optimized nanoscale phase separation of the PM6:L8‐BO matrix [20, 21]. Previous proof‐of‐concept studies have validated this approach, for example, incorporating Ir(III) complexes as sensitizers into polymer‐fullerene blends produced concurrent enhancements in short‐circuit current density (*J_sc_*) and *V*_oc_, the *V*_oc_ enhancement was ascribed to reduced non‐radiative losses via the aforementioned luminescence cascade mechanism [22, 23]. Here, we introduce a dual strategy combining ternary host engineering with molecular PS to overcome the efficiency limits of conventional OSCs. Specifically, we designed and synthesized two novel, low‐cost pyrazine‐based donor polymers: poly[2‐(5‐(4,8‐bis(5‐(6‐((2‐hexyldecyl)oxy)naphthalen‐2‐yl)thiophen‐2‐yl)‐6‐methylbenzo[1,2‐b:4,5‐b']dithiophen‐2‐yl)‐4‐(2‐ethylhexyl)thiophen‐2‐yl)‐5‐(4‐(2‐ethylhexyl)‐5‐methylthiophen‐2‐yl)pyrazine] (denoted as PBDT‐Pyz or N1) and poly[2‐(5‐(4,8‐bis(5‐(6‐((2‐hexyldecyl)oxy)naphthalen‐2‐yl)thiophen‐2‐yl)‐6‐methyldithieno[2,3‐d:2',3'‐d']benzo[1,2‐b:4,5‐b']dithiophen‐2‐yl)‐4‐(2‐ethylhexyl)thiophen‐2‐yl)‐5‐(4‐(2‐ethylhexyl)‐5‐methylthiophen‐2‐yl)pyrazine] (denoted as PBDT‐T‐Pyz or N2). These polymers were designed with deep highest occupied molecular orbital (HOMO) levels and highly coplanar backbone structures to promote efficient charge transport and high *V_oc_*. Donors were incorporated into the benchmark PM6:L8‐BO host system, and this was followed by doping with a green‐emitting Ir‐3 complex as a PS. The resulting ternary active layer displayed an optimal morphology and well‐defined energy transfer pathways. The Ir‐3 complex plays a multifaceted role because it functions as (i) a spectral sensitization (SS), absorbing photons in the green‐to‐visible region and transferring energy to the host via TTET, (ii) a mediator in a *V*_oc_ enhancing luminescent cascade that suppresses non‐radiative recombination, and (iii) an interfacial passivator that improves charge collection efficiency. Accordingly, the champion device, based on the PM6:N2:L8‐BO blend containing 1 wt.% Ir‐3, achieved a record PCE of 18.6%, with a *V_oc_* of 0.88 V, *J_sc_* of 27.6 mA cm^−^^2^, and fill factor (FF) of 76.7%, enabled by a well‐defined TTET cascade (Ir‐3 (T_1_ = 2.46 eV) → L8‐BO (T_1_ = 1.85 eV), ΔET_1_ = 0.61 eV) that effectively funnels triplet excitons into productive charge generation while simultaneously suppressing non‐radiative *V_oc_* losses via a luminescent cascade respectively. This PCE represents a 2.6% absolute enhancement over the binary PM6:L8‐BO control (16.0%), signifying that synergistic ternary host engineering and phosphor sensitization provides a powerful, eco‐friendly, and halogen‐free solvent‐compatible pathway to exceed the performance limits of conventional OSCs.

## 2 Results and Discussion

### 2.1 Design Strategy, Synthesis, and Characterization

We designed and synthesized two D‐A type pyrazine‐based donor polymers, PBDT‐Pyz (N1) and PBDT‐T‐Pyz (N2), along with a green‐emitting Ir‐3 complex as a PS. N1 incorporates a pyrazine acceptor integrated into an ANPh‐conjugated benzo[1,2‐b:4,5‐b′] dithiophene (BDT) donor core, whereas N2 contains the same pyrazine acceptor with an ANPh‐conjugated dithieno[2,3‐d:2′,3′‐d′] benzo[1,2‐b:4,5‐b′] dithiophene (DTBDT) donor core (Figure 1a,b). Synthetic routes are detailed in Scheme S1 . N1 and N2 were prepared using Stille coupling reactions between the respective brominated pyrazine monomers and the corresponding stannylation BDT or DTBDT monomers [24, 25]. The Ir‐3 complex was synthesized according to Scheme S2 using a multi‐step process involving (i) nucleophilic substitution of 3‐amino‐2‐chloropyridine with 2‐ethylhexyl bromide to form 2‐chloro‐N‐(2‐ethylhexyl)pyridin‐3‐amine, (ii) benzoylation followed by cyclization to yield 5‐(2‐ethylhexyl)benzo[c][1,5]naphthyridin‐6(5*H*)‐one, (iii) the formation of the iridium chloro‐bridged dimer, and (iv) final substitution with 2‐picolinic acid to afford Ir‐3. The molecular structures of all monomers and intermediates were confirmed by ^1^H‐ and ^13^C‐NMR Figures S1‐S4, and the Ir‐3 complex was further characterized by high‐resolution mass spectrometry (Figure S5). The molecular weights of N1 and N2 were determined by gel permeation chromatography (GPC) using THF as eluent; number‐average molecular weights (*M_n_*), weight‐average molecular weights (*M_w_*), and polydispersity indices (PDIs) are summarized in Table S1 . Both polymers showed good solubility in common organic solvents (*o*‐xylene, toluene, chlorobenzene, and chloroform) Table S2. The design strategy ensures that N1 and N2 have deep HOMO levels and planar backbones for effective charge transport [26], and Ir‐3 provides strong SOC for triplet harvesting. Collectively, these features provide high‐performance PS‐OSCs [27, 28, 29, 30]. *J_sc_* (25.8 to 27.6 mA cm^−^^2^), *V_oc_* (0.85 to 0.88 V), and FF (73.0 to 76.7%) are all simultaneously improved by this combination, resulting in a best device PCE of 18.6%.

**FIGURE 1:**
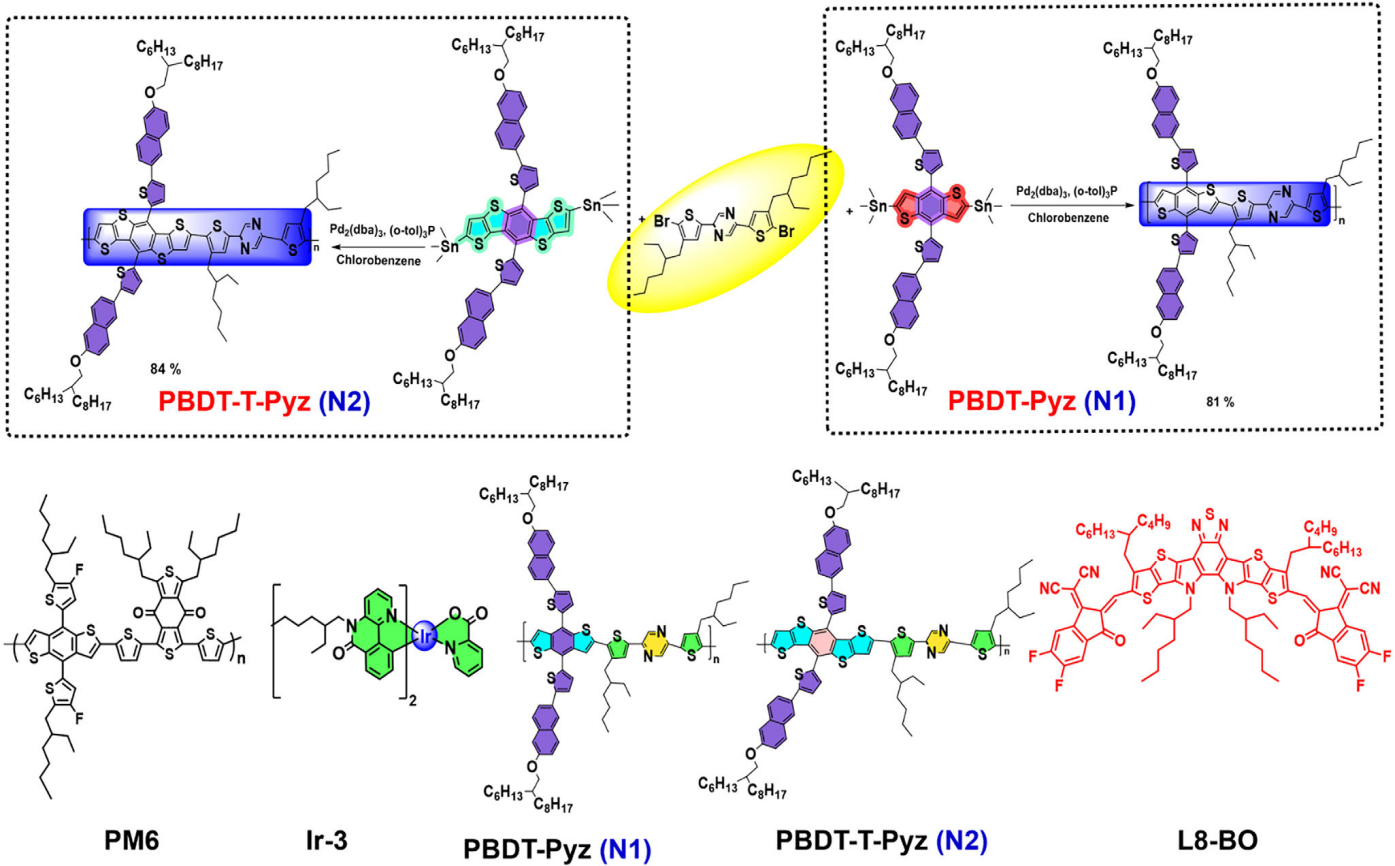
Synthetic route for wide bandgap donors. (a) PBDT‐T‐Pyz (N2), (b) PBDT‐Pyz (N1), (c) Molecular structures of PM6, PBDT‐T‐Pyz (N2), PBDT‐Pyz (N1), Ir‐3 complex, and L8‐BO.

### 2.2 Optical, Electrochemical, and Thermal Properties

Figure 2a shows the UV–vis absorption spectra of Ir‐3, PM6, L8‐BO, N1, and N2. N1 and N2 donor polymers showed broad absorption from 300 to 700 nm. PM6 and L8‐BO show their characteristic absorption in the visible range, indicating their suitability as host donor polymers in the ternary host system. As depicted in (Figure 2b) the energy levels of N1, N2, and Ir‐3 were estimated by cyclic voltammetry, as shown in Figures S6 and S7. The calculated HOMO/the lowest unoccupied molecular orbital (LUMO) energy levels of N1 and N2 were −5.59/−3.63 eV and −5.58/−3.60 eV, respectively. The E_g_^opt^ values of N1 and N2 were 1.96 eV and 1.98 eV in Table S3. As given in the Figure 2b, the alignment of cascade energy levels formed between the HOMOs and LUMOs of these active layer components improved the CT and collection capabilities by reducing the CT‐ barrier and therefore would benefit high *V_oc_* and enhance device performance. Ir‐3 complex possessed a HOMO/ LUMO of ‐5.5 eV/−3.0 eV with a large optical band gap (E_g_^opt^) of 2.50 eV, as given in Table S4. This energy level alignment creates a favorable cascade between PM6 (−5.50/−3.61 eV) and L8‐BO (−5.72/−3.90 eV), which supports smooth stepwise CT while minimizing energy losses. In addition, extensive exciton separation and charge extraction are facilitated via cascade‐like energetic alignment between N1/N2, PM6, and L8‐BO [31]. Furthermore, the Ir‐3 complex showed visible‐range absorption, which is essential for SS [32]. Importantly, in addition to its dominant ligand‐centered absorption in the UV region (300–400 nm), the green‐emitting Ir‐3 complex shows no resolvable secondary absorption band in the green‐to‐visible region (Figure 2a); any MLCT contribution in this region is too weak to be distinguished from the baseline. Its phosphorescence, however, spans 470–700 nm with emission maxima (λmax) at 523 nm. This is complementary to that of the host and necessary for a primary role as a SS. Figure 2c shows that the UV–vis spectra of N1 and N2 polymers depict good absorption wavelength in the range 400–700 nm. N2 exhibited λ_max_ at 544 and 588 nm, while N1 showed λ_max_ at 540 and 588 nm, corresponding to the (0–1 peaks) and (0–0 peaks) associated with π‐π* vibronic transitions and intramolecular charge transfer (ICT) processes. Notably, N1 had a weaker absorption peak (0–0) than N2, suggesting N2 might aggregate strongly in solution. The aggregation properties of N2 and N1 were studied using variable temperature‐dependent UV‐vis absorption spectra from RT to 100°C, as shown in Figures S8 and S9. The A^0–1^/A^0–0^ vibronic shoulder peak of N2 partially faded at 100°C, whereas that of N1 did not, implying that N2 aggregates less at high temperatures. After cooling the N2 solution to RT, its absorption peak red‐shifted to 544 nm, reflecting strong aggregation and 𝜋–𝜋 stacking of N2 at RT. These observations demonstrated N2 is more aggregation sensitive to temperature than N1. The slightly larger E_g_^opt^ of N2 (1.98 eV) relative to N1 (1.96 eV), despite its red‐shifted absorption maximum, is attributed to the sharper absorption onset of N2 arising from its higher degree of molecular ordering and reduced energetic disorder. This behavior is consistent with the narrower density of states distribution observed in more crystalline conjugated polymers, wherein stronger intermolecular π‐π interactions simultaneously red‐shift the vibronic absorption maxima and steepen the band edge, resulting in a marginally larger on‐set derived optical bandgap [33]. This interpretation is further supported by density functional theory (DFT) calculations, which reveal nearly identical frontier orbital energies for N1 and N2 (HOMOs, –4.17 vs. –4.16 eV, LUMOs, −1.72 vs. −1.72 eV), which indicates that the observed E_g_^opt^ difference reflects solid‐state packing effects rather than intrinsic electronic structure differences. Furthermore, the thermal properties of N1, N2, and Ir‐3 were studied by thermogravimetric analysis (TGA) and differential scanning calorimetry (DSC) under nitrogen (N_2_) atmosphere, which showed that N1, N2, and Ir‐3 had exceptional thermal stability, as shown in Figure 2d and Table S3. TGA revealed that N1, N2, and Ir‐3 are thermally stable with 5% weight loss decomposition temperatures (T_d_) of 376, 379, and 362°C, respectively, as shown in Table 1, which showed they were thermally resistant and suitable for solar cell applications. While the Ir‐3, T_d_ (362°C) is lower than that of N1, T_d_ (376°C) and N2, T_d_ (379°C), all three components exhibit excellent thermal stability, suggesting that the pyrazine unit incorporation provides both donor polymers with high thermal stability. DSC measurements show no significant crystallization or melting peak in N1 and N2 polymers, along with Ir‐3 suggesting both polymer and Ir‐3 complex exhibit stable thermal behavior Figures S10 and S11. These results confirm the exceptional thermal stability of the synthesized materials, supporting their suitability for PS‐OSC applications [34, 35]. This result correlates with significant Ir(III)‐based PS‐OSC studies, which indicate that device performance is critically dependent on the molecular configuration of the PS, the donor polymers' preferred face‐on packing orientation, and the thermal stability of the active layer blend [36]. The normalized absorption spectra, as given in Figure S12, and the absorption coefficient spectra of binary and ternary blend films were shown in Figure 2e. The PM6:N2: L8‐BO ternary blends containing Ir‐3 showed stronger absorption than the binary host PM6: L8‐BO blend, indicative of improved light harvesting (LH) after incorporation of the pyrazine‐based donor and PS. The PM6:N2: L8‐BO blend with doped Ir‐3 had a greater absorption coefficient of (ε = 1.50 × 10^5^ cm^−1^) greater than PM6:N1: L8‐BO blend with doped Ir‐3 (ε = 1.37 × 10^5^ cm^−1^), suggesting that the N2‐based donor provides the most favorable optical environment for exciton generation and higher *J_sc_* and PCE values. Figure 2f depicts the ternary films' steady‐state photoluminescence (PL) spectra. By incorporating 1 wt.% Ir‐3, both sensitized blends showed improved PL‐quenching of the host emission (λem = 660–690 nm), with a weak shoulder feature at 780–820 nm. ΔPLmax = 94.03% for PM6:N1:L8‐BO and 98.86% for PM6:N2:L8‐BO as given in Table S5, exhibiting highly effective exciton dissociation and energy transfer mediated via the Ir‐3 sensitizer when compared to the unquenched host emission profiles of the Ir‐3 free blends shown in Figure S14. The host emission broadens and red‐shifts with Ir‐3 insertion, with the dominant peak shifting from 660–665 nm to 680–690 nm and an extra shoulder emerging near 730 nm, corresponding with the population of the L8‐BO triplet‐derived emission via Ir‐3‐mediated TTET. The stronger quenching observed for PM6:N2: L8‐BO containing Ir‐3 film suggests N2 promotes better interfacial excited‐state interactions than N1. This behavior is consistent with the concept that compatible Ir‐3 sensitizers can prolong exciton utilization and suppress recombination in ternary PS‐OSCs [37]. The triplet harvesting and energy transfer mechanism (Figure 2g) is a multi‐step energy recycling cascade that collects triplet excitons (T_1_) that would normally be lost via non‐radiative decay in conventional OSCs. On photoexcitation, the Ir‐3 sensitizer absorbs photons in the UV‐to‐visible region (350–530 nm), complementing the PM6:N2: L8‐BO host blend absorption profile and expanding the effective spectrum response, as indicated by the increased external quantum efficiency (EQE) in the 450–600 nm area (Figure 4c). The Ir(III) core strong SOC permits a near quantitative ISC, effectively converting the singlet excited state (S_1_) into a long‐lived ^3^MLCT triplet state (T_1_ = 2.46 eV), as confirmed by the structured 77 K phosphorescence emission at 505–520 nm (Figure 4d) [13, 14]. Triplet excitons are irreversibly transferred to L8‐BO via Dexter‐type TTET, thermodynamically driven by a favorable energy offset of ΔET_1_ = 0.61 eV, populating the L8‐BO T_1_ state (1.85 eV) as directly confirmed by the emergence of the 670 nm phosphorescence band in all blend films (Figure 4d) and enabling triplet exciton dissociation into free charge carriers at the D/A interface [15, 16]. Beyond spectral sensitization, Ir‐3 serves a multifunctional role: (I) as a mediator in a *V_oc_* enhancing luminescent cascade that suppresses nonradiative recombination losses, as evidenced by the reduced Plight vs. *V_oc_* slope (1.03 kT/q) (Figure 4h) [18, 19], and (ii) as an interfacial passivation that reduces trap‐state density (N_trap_ = 1.37 × 10^16^ cm^−^^3^) and charge transport resistance (RCT = 807.6 Ω cm^2^), thereby improving charge collection efficiency (Figures S23 and S24) [6, 38]. *J_sc_* (25.8 to 27.6 mA cm^−^^2^), *V_oc_* (0.85 to 0.88 V), and FF (73.0 to 76.7%) are all simultaneously improved by this combination, resulting in a PCE of 18.6% (Table 2).

**FIGURE 2:**
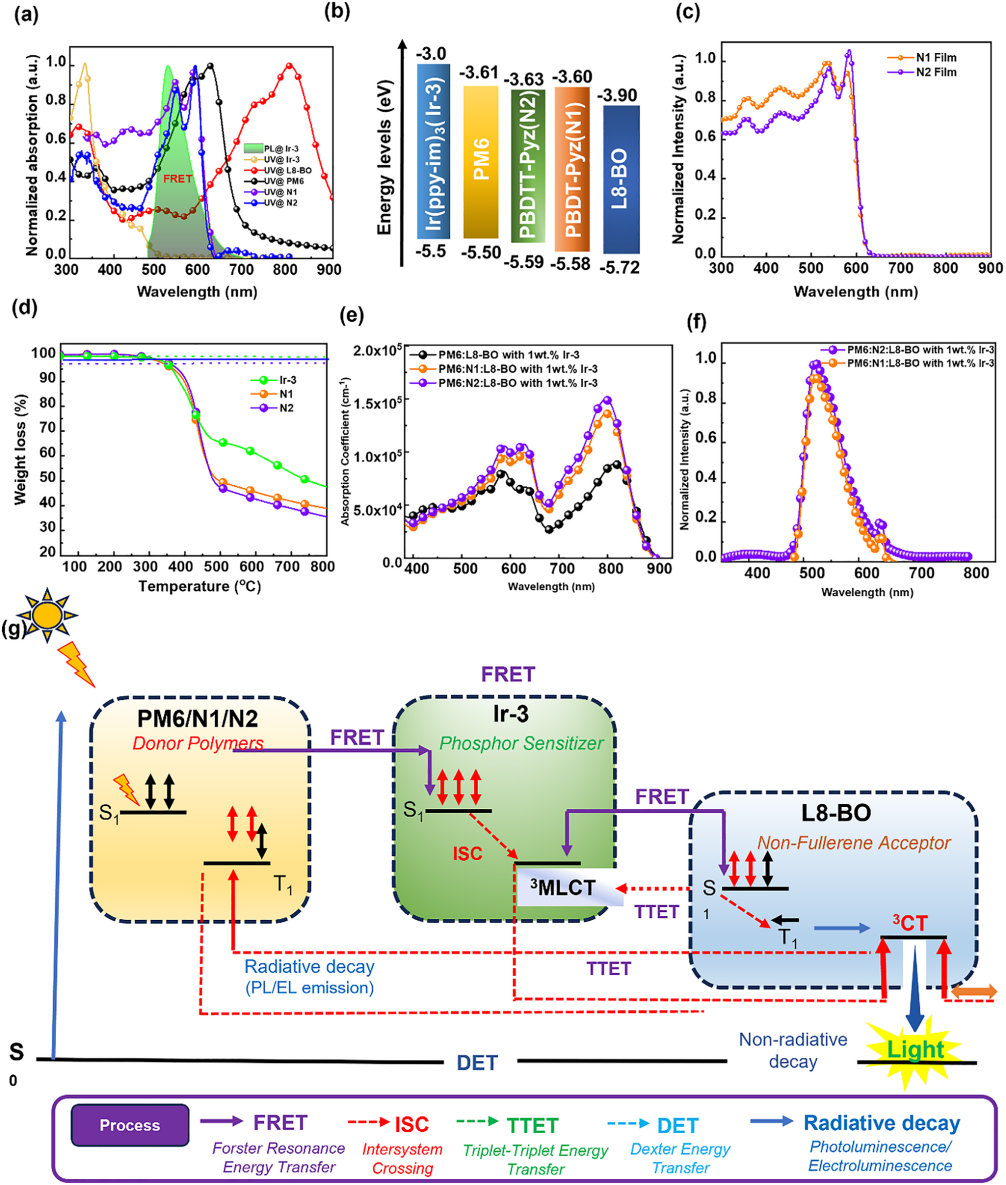
(a) UV‐vis spectrum of PM6, L8‐BO, N1, N2, Ir‐3, and PL of Ir‐3 complex. (b) Energy level diagram of Ir‐3, PM6, N1, N2 and L8‐BO. (c) UV‐vis spectrum of pyrazine‐based polymers in (i) films and (ii) in chloroform solutions. (d) TGA results for N1 and N2 polymer and Ir‐3 complex. (e) Absorption coefficient spectra of binary and ternary blend films of N1 and N2 doped with Ir‐3. (f) Steady‐state PL quenching spectra of PM6:N1: L8‐BO and PM6:N2: L8‐BO blend films with and without 1 wt.% Ir‐3. (g) Schematic illustration of FRET, ISC, TTET, and charge generation in the PM6/N1/N2–Ir‐3/L8‐BO device.

**TABLE 1:** Optical and electrochemical properties of N1, N2, and Ir‐3.

Polymer Donors	λ_max_ ^a^ [nm]	λ_max_ ^b^ [nm]	E_g_^opt^ ^c^ [eV]	HOMO^d^ [eV]	LUMO^e^ [eV]	T_d_ ^f^ [°C]
PBDT‐Pyz (N1)	540, 587	540, 588	1.96	−5.59	−3.63	376
PBDTT‐Pyz (N2)	544, 587	544, 588	1.98	−5.58	−3.60	379
Ir‐3	—	—	2.50	−5.50	−3.0	362

^a^ Measured in chloroform at 10^−5^ mol L^−1^

^b^ Measured as film

^c^ Optical band gaps (E_g_^opt^) were calculated from the onset of UV‐vis spectra, E_g_^opt^ = 1240/λ

^d^ HOMO values were determined by the onset of CV measurements, HOMO = ‐((E_ox_, _onset_− E_FC_/_FC+_) + 4.8)

^e^ LUMO values were calculated from HOMO values, LUMO = HOMO + E_g_^opt^

^f^ Temperature at a mass loss of 5% was measured by TGA using a heating rate of 10°C min^−1^ under N_2_.

**TABLE 2:** Summary of PS‐OPV performances under AM 1.5G at 100 mW cm^‒2^ illumination.

Active layer ^a^	*V*_oc_ ^b^ [V]	*J*_sc_ ^b^ [mA/cm^2^]	*J*_sc_ ^c^ [mA/cm^2^]	FF ^b^ [%]	PCE ^b^ [%]
PM6:L8‐BO	0.85 (0.82± 0.03)	25.8 (24.8± 1.0)	24.9	73.0 (72.8±0.2)	16.0 (15.97± 0.03)
PM6:L8‐BO with 1 wt. %Ir‐3	0.86 (0.84± 0.02)	26.5 (25.2± 1.3)	25.4	73.4 (72.8±0.6)	16.7 (16.67± 0.03)
PM6:N1: L8‐BO with 1 wt. %Ir‐3	0.87 (0.86± 0.01)	27.3 (26.5± 0.8)	26.1	74.2 (73.9±0.3)	17.6 (17.58± 0.02)
PM6:N2: L8‐BO with 1 wt. %Ir‐3	0.88 (0.87± 0.01)	27.6 (27.0± 0.6)	26.5	76.7 (76.5±0.2)	18.6 (18.59± 0.01)

^a^ Blend films were processed using o‐xylene and annealed at 100°C for 10 min.

^b^ An average of 20 devices were evaluated.

^c^ Obtained by integrating EQE spectra.

### 2.3 Computational Analysis of the Molecular Geometry and Electronic Structure of PS‐OSCs

To gain insight into the charge generation process, we performed DFT calculations (B3LYP/6‐31G*) of N1, N2, and Ir‐3 segments to examine the molecular geometry and electronic structure of N1, N2, and Ir‐3, providing insight into their suitability for PS‐OSCs. As shown in Figure 3a–c, both N1 and N2 have long conjugated backbones with delocalized frontier orbitals, which are beneficial for intramolecular charge transport (ICT). The computed molecular electrostatic potential (ESP) surface, and HOMO/LUMO energies of all three components are presented in Figure 3a–c. Both N1 and N2 adopt highly planar backbone conformations with delocalized frontier orbitals extending across the full conjugated repeat unit, which is favourable for efficient ICT. The top and side view of N1 and N2 polymers are summarised in Figures S15 and S16. The DFT‐calculated HOMO/LUMO levels are −4.17/−1.72 eV for N1 and −4.16/−1.72 eV for N2 intrinsic electronic structure. Despite this similarity in frontier orbital energies, N2 exhibits a higher dipole moment (*µ* = 1.40 D) relative to N1 (*µ* = 0.94 D), indicating greater intramolecular charge asymmetry and a more polarized electronic distribution. This enhanced polarization in N2 is expected to promote stronger intermolecular electrostatic interactions and a more ordered solid‐state packing arrangement, consistent with the superior grazing‐incidence wide‐angle x‐ray scattering (GIWAXS) coherence lengths and reduced π‐π stacking distance (*d*π = 3.55 Å vs. 3.62 Å for N1) observed experimentally. The DFT‐calculated backbone dihedral angles Q1‐ Q6 for both polymers remain within a narrow range Table S6, confirming a rigid and conformationally stable π‐framework that allows efficient charge delocalization and minimizes energetic disorder in the solid state. The slightly larger Q6 torsion angle in N2 contributes to its more favorable intermolecular packing and the superior device performance of N2‐based PS‐OSCs. The most pivotal aspect of the computational analysis concerns the electronic structure of the Ir‐3 complex and the mechanistic basis of its ^3^MLCT excited state, which underpins its function as a phosphorescent sensitizer. As illustrated in Figure 3c, the HOMO and LUMO of Ir‐3 exhibit markedly distinct spatial distributions that are characteristic of a mixed MLCT/ligand‐to‐ligand charge transfer (LLCT) electronic transition. The HOMO is localized on the Ir metal center (d*π* (Ir)) and the electron‐rich π system of the cyclometalating benzo[*c*] [1, 5] naphthyridinyl chromophore (the *N*‐heterocyclic donor unit), reflecting the d*π*(Ir) character of the highest occupied state. In contrast, the LUMO resides on the π*‐antibonding orbitals of the 2‐picolinate ancillary ligand and the electron‐deficient naphthyridinone moietyof cyclometalating units. Therefore, photoexcitation of Ir‐3 drives electronic density from the metal‐centered d*π*(Ir) orbital to the ligand‐centered π*(L) orbital, constituting a canonical d*π*(Ir) → π*(L) MLCT transition. This spatial charge redistribution following excitation is visually apparent from the complementary HOMO and LUMO isosurface plots in Figure 3c, where the electron density clearly migrates from the metal core toward the electron‐withdrawing *N*‐heterocyclic ligand fragment after excitation. This behavior is directly analogous to the MLCT mechanism established for archetypal *fac*‐Ir(III) cyclometalated complexes such as *fac*‐Ir(ppy)_3_ and *fac*‐Ir(tBufppy)_3_, in which the HOMO resides predominantly on the Ir center and the π‐donating phenyl unit, while the LUMO is localized on the π‐accepting pyridyl fragment.

**FIGURE 3:**
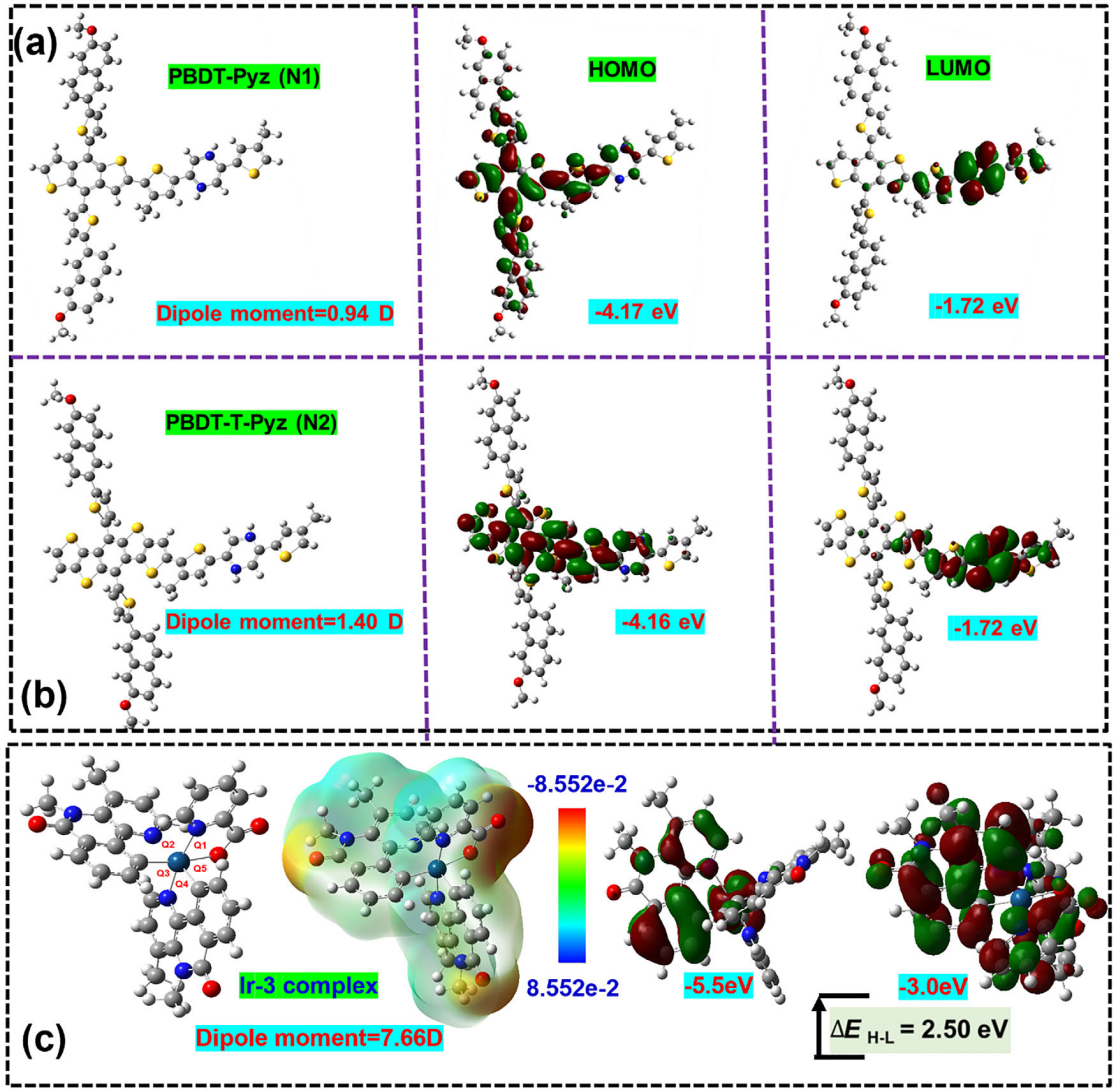
DFT analysis showing (a) top view with HOMO/LUMO energies of N1, (b) top view of HOMO/LUMO energies of N2, and (c) top view with ESP and HOMO/LUMO energies of the Ir‐3 complex.

### 2.4 Photovoltaic Device Performance of PS‐OSCs

To determine the effectiveness of the phosphor sensitization strategy (PSS), OSCs with an inverted structure, including ITO/ZnO/Active Layer/MoO_3_/Ag, were designed (Figure 4a). The active layers were obtained with a green solvent (*o*‐xylene) and, based on the optimization; the doping concentration of the Ir‐3 complex was set to 1 wt.%. The optimization involved adjusting the donor‐acceptor (D/A) ratio and the thermal annealing temperature with a 1,8‐diiodooctane (DIO) additive and using different solvent processing conditions. The optimal annealing condition for dual‐solvent‐processed PS‐OSC devices was optimized at 100°C/10 min. Figures S16–S20, which evaluated Ir‐3 doped N1 and N2‐based binary, N1 and N2 ternary devices without Ir‐3, solvent effects, thickness, and blend ratios, are provided in Tables S9‐S13. The molecular weights of the N2 and N1 polymers and Ir‐3 (283450, 200000, and 930.35 g mol^−1^, respectively) played a crucial role in chain entanglement due to attached pyrazine‐π‐conjugated groups, as listed in Table S1. Figure 4b illustrates the current density–voltage *(J–V)* parameters of the PM6:N2: L8‐BO blend with doped Ir‐3, as shown in Table 2. The binary PM6:L8‐BO host device produced an FF of 73.0%, PCE of 16.0%, *V*_oc_ of 0.85 V, and *J_sc_* of 25.8 mA cm^−^^2^. With *V*_oc_ of 0.87 V, *J_sc_* of 27.2 mA cm^−^^2^, and FF of 74.1%, the PM6:N2:L8‐BO device without Ir‐3 achieved a PCE of 17.5%, whereas the PM6:N1:L8‐BO device without Ir‐3 obtained a PCE of 17.0%. In contrast to the improvement from 16.0% (PM6:L8‐BO) to 17.5% (PM6:N2:L8‐BO without Ir‐3), which was attributed to complementary absorption and cascade energy alignment as shown in Figure S17 and Table S10, the additional improvement from 17.5% to 18.6% with Ir‐3 incorporation led to an absolute gain of 1.1% that can be directly attributed to the phosphor‐sensitization effect. Additionally, the best device, which achieved a PCE of 18.6%, *V*_oc_ of 0.88 V, *J_sc_* of 27.6 mA cm^−^^2^, and FF of 76.7%, was fabricated via the addition of 1 wt.% Ir‐3 to the PM6:N2: L8‐BO ternary blend. On the other hand, the N1‐based ternary device similarly showed significant improvement, obtaining a PCE of 17.6% (Table 2). These results align in support of recent research showing that triplet sensitizers simultaneously boosted PS‐OSCs, *J_sc_*, and *V*_oc_ values by enhancing spectral amplification and reducing non‐radiative recombination losses [3, 19, 39]. The sensitization effects were explicitly shown via EQE spectra (Figure 4c). With a maximum EQE of more than 85%, the best device exhibited the broadest and most efficient photoresponse across the 400–900 nm range. In addition, an increased EQE in the 450–600 nm range coincided with the Ir‐3 absorption profile, indicating that Ir‐3 contributes to photocurrent generation via the triplet‐harvesting mechanism. Similarly, the precision of the photovoltaic results was verified by integrated *J_sc_* values which fitted the *J–V* measurements within ±5%. This SS effect, achieved via the PSS strategy involving heavy metal complexes that extend the photoresponse into the green‐to‐visible region, is comparable to previously reported approaches [15, 16]. Furthermore, to explain the TTET mechanism, phosphorescence spectra were recorded at 77 K for neat Ir‐3 and blend films (Figure 4d). The neat Ir‐3 complex displays a structured phosphorescence emission with a vibronic progression, exhibiting prominent peaks at approximately 505 and 520 nm, from which the E_T1_ of Ir‐3 is estimated to be 2.46 eV based on the high‐energy onset of the 0‐0 vibronic component. This well‐resolved vibronic fine structure is characteristic of cyclometalated Ir(III) complexes possessing mixed ^3^MLCT/^3^LC triplet excited‐state character, wherein strong SOC at the iridium center enables highly efficient ISC [13, 40]. Crucially, in all ternary and binary blend films examined (PM6:Ir‐3:L8‐BO, PM6:N1:Ir‐3:L8‐BO, PM6:N2:Ir‐3:L8‐BO), the characteristic Ir‐3 phosphorescence bands at 505–520 nm are strongly quenched, with PL quenching efficiencies of 94.03% and 98.86% for the N1‐ and N2‐based ternary blends, respectively (Table S5), and a sharp emission band emerges at 670 nm, consistent with the triplet excited state of the L8‐BO non‐fullerene acceptor (E_T1_ = 1.85 eV) with vibronic progression at 800 nm [41]. The near‐complete quenching of Ir‐3 triplet emission provides strong spectroscopic evidence for highly effective and unidirectional TTET from the Ir‐3 sensitizer to L8‐BO, thermodynamically driven by a substantial energy offset of ΔE_T1_ = 0.61 eV (E_T1_ of Ir‐3‐ E_T1_ of L8‐BO i.e., 2.46‐1.85 eV). This sizeable driving force ensures both the thermodynamic favourability and kinetic irreversibility of the TTET process, funneling triplet excitons into productive charge generation [42]. The phosphorescence profiles of the N1 and N2‐containing ternary blends containing Ir‐3 are nearly coincident with the binary PM6:Ir‐3:L8‐BO reference, confirming that N1 and N2 do not perturb the Ir‐3→L8‐BO TTET pathway, and instead function primarily at the upstream singlet exciton harvesting stage, facilitating Förster energy transfer (FRET) or direct ISC sensitization on the PM6 donor [15, 19]. A distinct and energetically well‐defined triplet exciton cascade PM6 (S_1_) → Ir‐3 (T_1_, 2.46 eV) → L8‐BO (T_1_, 1.85 eV) is determined based on combined 77 K phosphorescence data. Also, to understand the underlying charge carrier dynamics and recombination kinetics, time‐resolved photoluminescence (TRPL) measurements were performed. The TRPL decay curves for the Ir‐3‐doped ternary blend films (Figure 4e) exhibited non‐trivial decay kinetics, the TRPL decay curves were fitted using a bi and tri‐exponential decay function: I(t) = A^1^exp(‐t/τ^1^) + A^2^exp(‐t/τ^2^) + A^3^exp(‐t/τ^3^), with adjusted R^2^ > 0.998 for all fits (Table S14). The fast component (τ^1^) is attributed to prompt singlet exciton dissociation at D/A interfaces, the intermediate component (τ^2^) corresponds to the Ir‐3‐mediated ISC and subsequent TTET process, which introduces a transient exciton reservoir that temporarily sustains the PL intensity before energy is transferred to L8‐BO, and the slow component (τ^3^) arises from delayed emission via triplet‐derived charge recombination and exciton diffusion from the bulk to D/A interfaces. The shoulder feature originates specifically from the interplay between τ^2^ and τ^3^ and the ISC/TTET pathway transiently feeds the emissive population, before the TTET depopulation pathway dominates (producing the resumed decay).For the Ir‐3‐free ternary blends (Figure S21), the TRPL decay without Ir‐3 was adequately described by a bi‐exponential model, with average lifetimes (τ_avg_) of 1.27 ns for PM6:N1:L8‐BO and 1.12 ns for PM6:N2:L8‐BO, reflecting prompt dissociation and bulk diffusion only. Upon incorporation of 1 wt.% Ir‐3, the intensity‐weighted τ_avg_ decreased to 0.88 ns for the N1‐based Ir‐3‐doped ternary blend and 0.58 ns for the N2‐based Ir‐3‐doped ternary blend (Figure 4e and Table S14). The progressive reduction from the N1‐based to the N2‐based system is consistent with the superior morphological compatibility of N2 consisting of finer fibrillar network, lower trap density, which facilitates closer Ir‐3‐L8‐BO proximity and faster TTET. Thus, it implies that PS facilitates rapid TTET, which in turn suppresses geminate recombination and enhances charge generation efficiency [17, 18]. The effects of PS on hole and electron charge carrier mobilities (µ_h_ and µ_e_) were measured using the space‐charge‐limited current (SCLC) method for binary and ternary PS‐OSCs (Figure 4f). µ_h_/µ_e_ values for PM6:L8‐BO, PM6:N1:L8‐BO, and PM6:N2:L8‐BO were 5.03 × 10^−4^/4.49 × 10^−4^ cm^2^ V^−1^ s^−1^, 6.52 × 10^−4^/6.15 × 10^−4^ cm^2^ V^−1^ s^−1^, and 6.45 × 10^−4^/6.26 × 10^−4^ cm^2^ V^−1^ s^−1^, respectively in Table S7. In addition, the best‐sensitized N2‐based device (PM6:N2:L8‐BO blend with 1 wt.% Ir‐3) had a maximum PCE of 18.6% and displayed the most balanced charge carrier mobility (µ_h_/µ_e_ = 1.03), which was closer to unity than those of binary (1.12) and N1‐based (1.06) ternary devices. The high FF of 76.7% is directly attributable to this balanced charge transport. High‐performance OSCs are distinguished having balanced charge transport, a vital aspect of PSS design [43, 44]. The dark *J‐V* study of the PS‐OSCs shows suppressed non‐radiative recombination. The lower dark current density (*J_d_*) of N2 (1.97  × 10^−6^ mA cm^−2^) than N1 (5.87 × 10^−6^ mA cm^−2^) as depicted in Figure S22 of ternary PS‐OSCs at 0 V indicates excellent charge transport properties, which enhance light absorption and charge carrier efficiency, and thus, increase *J*_sc_ values [45]. These results demonstrate simultaneous improvements in FF, *J_sc_*, and overall PCE. The lower N_trap_ of N1 and N2 ternary blend devices were 1.67 × 10^16^ cm^−3^, and 1.37 × 10^16^ cm^−3^, respectively (Figures S23 and S24) [5]. in The lower N_trap_ value of the N2 PS‐OSC device implies significantly greater exciton dissociation and better CT. PS‐OSCs are effective in promoting delocalized polaron generation and enhanced CT and exciton dissociation at the PM6/L8‐BO interface. Furthermore, as given in Figure S25 impedance spectra of the N2 (807.6 Ω cm^2^) based ternary PS‐OSC device showed it had a lower charge transport resistance (R_CT_) than the N1 and binary PM6:L8‐BO optimized 1434.6 and 1902.9 Ω cm^2^ devices. This may have been due to reduced charge recombination within the active layer and at interfaces in the ternary film, resulting in FF and *J*_sc_ increases. In addition, the slope of the light intensity (*P*_light_) vs. *J_sc_* [*P*_light_ (*J*_sc_ ∝P*light* ^α^)] plot for N2 was linear, indicating that bimolecular recombination was more effectively suppressed in N2 (α  =  0.98) than N1 (α  =  0.96) based PS‐OSC devices (Figure 4g). Also, *P*_light_ vs. *V_oc_* [*V_oc_* ∝ n(kT/q) ln (*P*_light_)] plots for N2 and N1‐based ternary PS‐OSC devices had slopes (α) of 1.03 and 1.12 kT/q, respectively (Figure 4h). To investigate exciton dissociation behavior in the optimized PS‐OSCs, the correlation between effective voltage (*V_eff_*) and photocurrent density (*J_ph_*) was examined for each ternary blend PS‐OSCs. PM6: N2:L8‐BO with Ir‐3 (97.3) was found to have a greater exciton dissociation probability *P (E, T)* than PM6: N1:L8‐BO with Ir‐3 (92.7) devices as depicted in the Figure 4i and Table S8, indicating efficient exciton dissociation at the active layer interfaces of the N2‐based device due to higher *J_sc_* and FF values. The smaller slope of the N2 based ternary device indicates suppressed trap assisted recombination losses, and therefore more effective charge transport with fewer morphological traps, indicating that introduction of the PS passivates trap states and reduces non‐radiative voltage losses [6, 12]. Altogether, these findings indicate that the combination of ternary host engineering and the Ir‐3 complex, PS, provides a powerful pathway to high‐performance PS‐OSCs with enhanced *J_sc_*_,_ *V_oc_*, and FF values.

**FIGURE 4:**
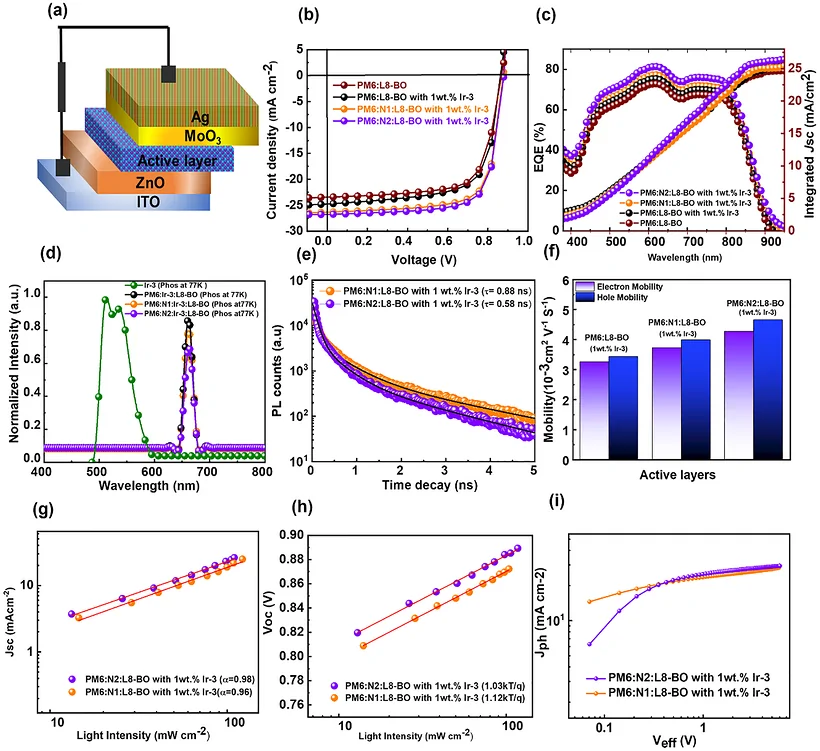
(a) Inverted device structure. (b) *J‐V* plots of PM6:L8‐BO, PM6: L8‐BO with1wt. % of Ir‐3, PM6:N1: L8‐BO with 1 wt. % of Ir‐3, and PM6:N2: L8‐BO with 1 wt.% of Ir‐3. (c) EQE plots of corresponding binary and N1 and N2‐based ternary devices. (d) 77 K phosphorescence spectra (λex = 380 nm) of neat Ir‐3 film and quaternary blend films (PM6: Ir‐3: L8‐BO, PM6:N1: L8‐BO with Ir‐3, PM6:N2: L8‐BO with Ir‐3), demonstrating highly efficient TTET cascade: Ir‐3 (T_1_ = 2.46 eV, 505–520 nm) → L8‐BO (T_1_ = 1.85 eV, 670 nm), with a vibronic progression at ∼800 nm. (e) TRPL decay curves of N1 and ‐based Ir‐3‐doped ternary blend films with solid tri‐exponential fitting. (f) Mobility µ_h_/µ_e_ ratio. (g) *J_sc_* vs. *P*_light_ intensity plots, (h) *V_oc_* vs. *P*_light_ plots. (i) J_ph_ vs. V_eff_ plots of N1 and N2‐based ternary PS‐OSCs.

### 2.5 Transient Absorption Spectroscopy (TAS) Analysis of Charge Dynamics

To further examine the photophysical processes underlying the enhanced performance of PS‐OSCs, we performed femtosecond TAS on pristine and Ir‐3‐doped blend films using two complementary excitation wavelengths: 605 nm, which falls within the absorption band of PM6, and 403 nm, which selectively excites the polymer donors (Figure 5). Because the Ir‐3 complex is present at only 1 wt.% of the total active layer and shows no resolvable absorption feature beyond its UV‐region ligand‐centered band (Figure 2a), any weak MLCT contribution is expected to be too small for Ir‐3‐specific spectral features to be cleanly resolved from the 2D‐TAS spectra at this loading. We therefore focus on kinetic differences between undoped and Ir‐3‐doped blends, which provide robust, spectrally‐independent evidence for sensitizer‐mediated processes. TAS signals are assigned as follows: GSB at 500–650 nm to PM6 and at 680–850 nm to L8‐BO, consistent with their absorption profiles in Figure 2a; and PIA at 850–950 nm to interfacial polaron formation. No resolvable signal was observed below 500 nm in either the binary or ternary blends at either excitation wavelength. Under 605 nm excitation, the binary PM6:L8‐BO film (Figure 5a,d,g,j) showed the expected PM6 GSB and polaron PIA features [11, 16, 17]. Ir‐3 doping left the overall spectral shape essentially unchanged but consistently slowed the 500–650 nm GSB recovery first in the binary blend, and further still in the ternary blends, with the N2‐based ternary film (Figure 5c,f,i,l) recovering more slowly than both the binary and N1‐based ternary films. This progression directly tracks the TRPL results: the N2‐based Ir‐3‐doped ternary blend has both the shorter average lifetime (τ_avg_ = 0.58 ns vs. 0.88 ns for N1) and the higher PL‐quenching efficiency (98.86% vs. 94.03%), indicating a faster, more efficient ISC/TTET process in this system. Because GSB recovery (TAS) and PL decay (TRPL) report on the same excited‐state population, the TAS‐derived recovery order (PM6:L8‐BO < PM6:N1:L8‐BO < PM6:N2:L8‐BO) and the TRPL‐derived lifetime/quenching order align, with two independent techniques supporting the same ISC/TTET pathway. Under 403 nm excitation, which selectively populates polymer‐donor singlet excitons (Figure 5g–i), Ir‐3‐containing blends again recombined more slowly than their undoped counterparts, consistent with an additional exciton‐utilization channel: FRET from PM6 to Ir‐3 (PM6 (S1) → Ir‐3 (S1)), followed by ISC and TTET to L8‐BO (Ir‐3 (S1) → Ir‐3 (T1) → L8‐BO (T1)). The N2‐based ternary blend showed the slowest recombination of all films examined, with GSB signals persisting beyond 5 ns. Although the low Ir‐3 loading prevents direct spectral observation of the Ir‐3 ^3^MLCT state, the systematic kinetic trends, namely slower GSB recovery, enhanced charge generation, and prolonged excited‐state lifetimes in Ir‐3‐containing blends, are fully consistent with the proposed ISC/TTET pathway and cannot be explained by morphology alone, since the Ir‐3‐free ternary blends (Figure S17 and Table S10) show none of these kinetic features [3, 4, 6, 19]. As a further, independent check of the ternary films, the Ir‐3‐free PM6:N1:L8‐BO and PM6:N2:L8‐BO films show TRPL τ_avg_ of 1.27 ns and 1.12 ns (Figure S21) markedly longerr than the ISC/TTET‐shortened decays of the corresponding Ir‐3‐doped ternary blends (Figure 4e and Table S14) reproducing, at the ternary level, the same TAS/TRPL correlation established above for the Ir‐3‐doped series. This use of TRPL as a kinetic cross‐check to transient‐absorption data follows established practice in triplet‐sensitized organic photovoltaic and phosphorescent host–guest systems, where Dexter‐type TTET through an intermediate, sensitizer‐localized reservoir state is characterized primarily by time‐resolved emission and corroborated by, rather than exclusively established through, TAS. Taken together, the TAS (Figure 5) and TRPL (Figure 4e and Figure S21) datasets are fully consistent with, and mechanistically corroborative of, the ISC/TTET pathway that slows excited‐state recombination across both binary and ternary blends a pathway established independently by 77 K phosphorescence and TRPL (Figure 4d,e).

**FIGURE 5:**
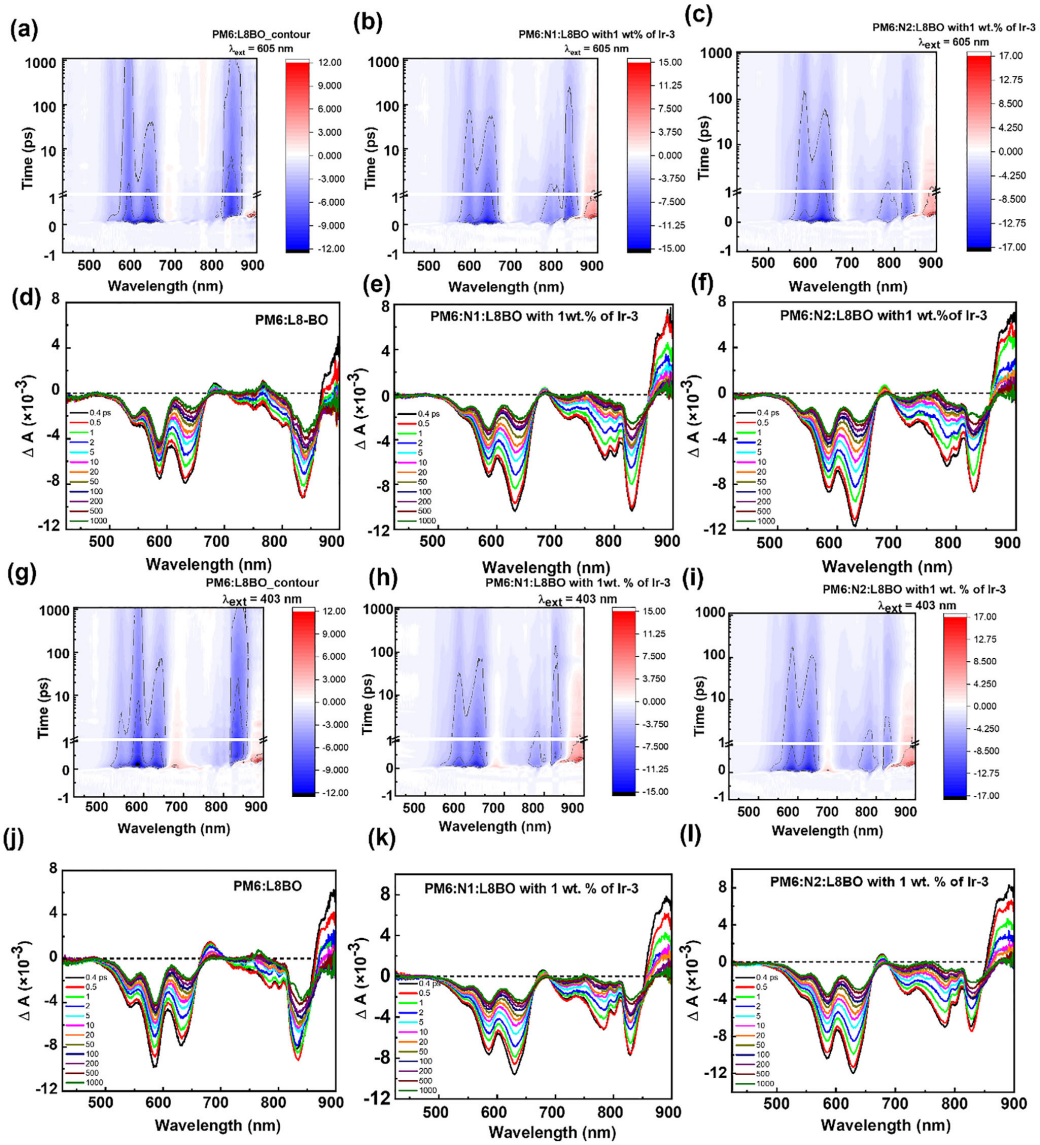
TAS images at an excitation wavelength of 605 nm (a‐f) for PM6:L8‐BO, PM6:N1: L8‐BO with 1 wt.% of Ir‐3, and PM6:N2: L8‐BO with 1 wt.% of Ir‐3. TAS images at an excitation wavelength of 403 nm (g‐l) for PM6:L8‐BO, PM6: L8‐BO with 1 wt.% of Ir‐3, PM6:N1: L8‐BO with 1 wt.% of Ir‐3, and PM6:N2: L8‐BO with 1 wt.% of Ir‐3.

### 2.6 Morphological Analysis of the Active Layer of PS‐OSCs Using GIWAXS

Grazing‐incidence wide‐angle x‐ray scattering (GIWAXS) was used to investigate the molecular packing and crystalline ordering of the active layers. The 2D GIWAXS patterns and corresponding line‐cut profiles of the neat and blend films are presented in Figure 6di–iv. Both N1 and N2 polymer donors adopted a preferential face‐on molecular orientation relative to the substrate, as evidenced by a prominent (010) *π‐π* stacking diffraction peak in the out‐of‐plane (OOP) direction at *q_z_* ≈ 1.6 Å^−1^ and a (100) lamellar stacking peak in the in‐plane (IP) direction at *q_xy_* ≈ 0.3 Å.^−1^[4, 6]. In this inverted device architecture, this face‐on orientation is essential for proper vertical CT. The molecular ordering of the two polymer donors differed significantly, as revealed by quantitative comparison of the (010) diffraction peaks. The N2 polymer exhibited a narrower and more intense (010) peak, corresponding to a smaller *π‐π* stacking distance (*d_π_* = 3.55 Å) compared to N1 (*d_π_* = 3.62 Å), indicating tighter intermolecular *π‐π* stacking and stronger electronic coupling between the conjugated backbones. The crystal coherence length (CCL), calculated from the full‐width at half‐maximum (FWHM) of the (010) peak using the Scherrer equation, was also larger for N2 (CCL = 94 Å) than for N1 (CCL = 75 Å), confirming a higher degree of long‐range molecular ordering in the N2 polymer. These observations are consistent with the higher dipole moment of N2 (*µ* = 1.40 D) compared to N1 *µ* = 0.94 D), which promotes stronger intermolecular electrostatic interactions and more ordered solid‐state packing. With respect to the binary PM6:L8‐BO and the N1‐based, the N2‐based ternary blend retained its preferable face‐on orientation with sharper and more distinct diffraction features after blending with PM6 and L8‐BO in Figure 6. Furthermore, the incorporation of 1 wt.% Ir‐3 into the N2 ternary blend further enhanced the molecular ordering, as evidenced by the 2D GIWAXS patterns shown in Figure S26. The *π–π* stacking distance was reduced to *d_π_* = 3.52 Å, and the CCL increased to 110 Å, representing a significant improvement over the undoped ternary blend. This result suggests that the Ir‐3 complex acts as a morphological compatibilizer, intercalating between the polymer chains and promoting tighter π‐π stacking without disrupting the favorable face‐on molecular orientation [38]. The enhanced molecular ordering and packing in the N2‐based Ir‐3‐doped ternary blend directly correlated with superior device performance. The best device achieved a high hole mobility of 6.45 × 10^−4^ cm^2^ V^−1^ s^−1^, a balanced charge transport ratio (µ_h_/µ_e_) of 1.03, and FF of 76.7%. The reduced *π‐π* stacking distance enables efficient charge carrier hopping between the conjugated backbones, while the face‐on orientation ensures vertical charge transport toward the electrodes, thereby minimizing bimolecular recombination losses [2]. Thus, the N2‐based ternary device demonstrated a benchmark PCE of 18.6% and surpassed its N1‐based counterpart in terms of molecular packing characteristics.

**FIGURE 6:**
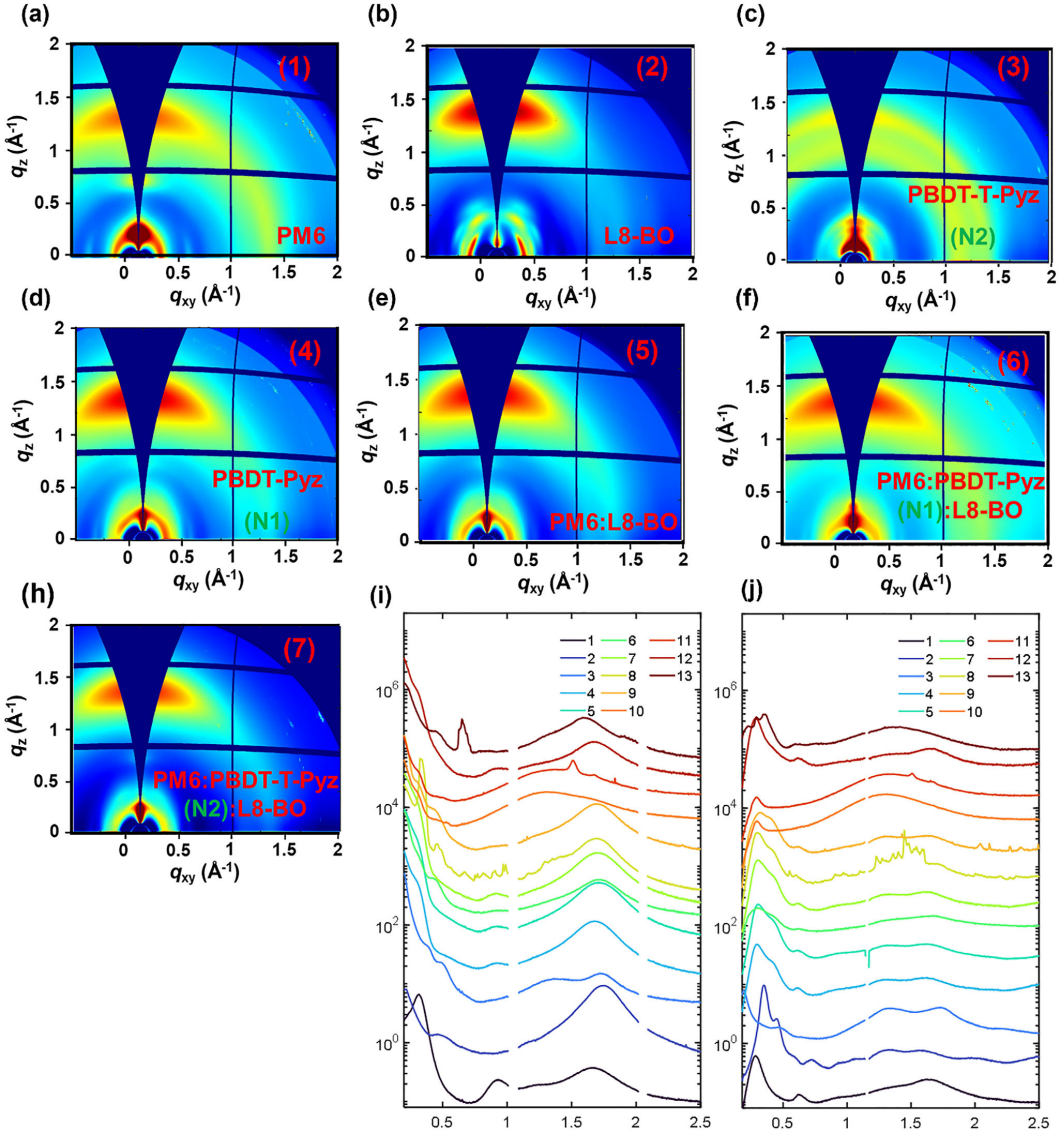
2D GIWAXS patterns of (a) PM6, (b) L8‐BO, (c) PBDT‐T‐Pyz (N2), (d) PBDT‐Pyz (N1), (e) PM6:L8‐BO, (f) PM6:N1: L8‐BO, (g) PM6:N2: L8‐BO, and (h) PM6:N2: L8‐BO doped with 1 wt. % Ir‐3. (i, j) Corresponding 1D out‐of‐plane and in‐plane GIWAXS line‐cut profiles, respectively.

### 2.7 AFM and TEM Analysis of the Active Layer Morphology

The surface and bulk morphologies of the active layers were characterized by atomic force microscopy (AFM), transmission electron microscopy (TEM), and 3D laser confocal microscopy (3D‐CLSM) (Figure 7). AFM height images (Figure 7a) revealed that the binary PM6:L8‐BO film had a relatively rough surface with a root‐mean‐square (RMS) roughness of 1.58 nm (Figure 7a‐i). With the incorporation of the N1 polymer donor and 1 wt.% Ir‐3 to form the ternary blend, the surface became smoother with an RMS roughness of 1.36 nm (Figure 7a‐ii). The N2‐based ternary blend (PM6:N2:L8‐BO with 1 wt.% Ir‐3) had the smoothest surface, with an RMS roughness of 1.03 nm (Figure 7a‐iii), indicating that the N2 polymer donor promoted superior component intermixing and that Ir‐3 functions as an effective morphological compatibilizer [6]. Complementary TEM imaging (Figure 7b) provided direct visual evidence of the nanoscale bulk morphology and D/A phase separation. The binary PM6:L8‐BO blend (Figure 7b‐i) revealed coarse, not well‐defined domains with poor interconnectivity, which detrimentally affects charge transport. In contrast, the N1‐based ternary blend containing Ir‐3 (Figure 7b‐ii) displayed a finer and more bicontinuous phase separation with individual fibril widths of approximately 15–25 nm. The N2‐based ternary blend (Figure 7b‐iii) exhibited the most well‐developed fibrillar network architecture, with individual fibril widths of the approximately 10–20 nm, which is well depicted within the typical exciton diffusion length of organic semiconductors range (10‐20 nm), thereby ensuring efficient exciton dissociation at the D/A interfaces [3]. To characterize morphological features at larger length scales, 3D‐CLSM was employed (Figure 7c). The domain spacing values (d = 120, 187, and 191 nm) for the binary, N1, and N2‐based ternary blends, respectively, refer to the long‐period surface texture periodicity, which represents the center‐to‐center distance between periodic surface features, not the individual D/A domain size. The surface area roughness (S_a_) simultaneously decreased from 6 nm (PM6:L8‐BO) to 5 nm in N1 to 4 nm in N2‐based ternary film with Ir‐3 doping. The progressive increase in surface periodicity accompanied by decreasing S_a_ indicates that N2 incorporation drives a more ordered and uniform phase separation with smoother interfacial morphology. Importantly, the high FF of 76.7%, and PCE of 18.6% are consistent with the nanoscale morphology observed by TEM, where the actual D/A phase separation occurs at 10–20 nm length scales, not at the larger 191 nm periodicity measured by 3D‐CLSM. The improved long‐range morphological ordering, combined with the ns bicontinuous fibrillar network, directly promotes the TTET process by ensuring closer proximity between Ir‐3 sensitizer molecules and L8‐BO acceptor domains, thereby reducing the triplet exciton diffusion distance required for productive energy transfer and contributing to the superior PL quenching efficiency (98.86%), high FF of 76.7%, balanced charge carrier mobility (µ_h_/µ_e_ = 1.03), and PCE of 18.6% of the N2‐based device [39].

**FIGURE 7:**
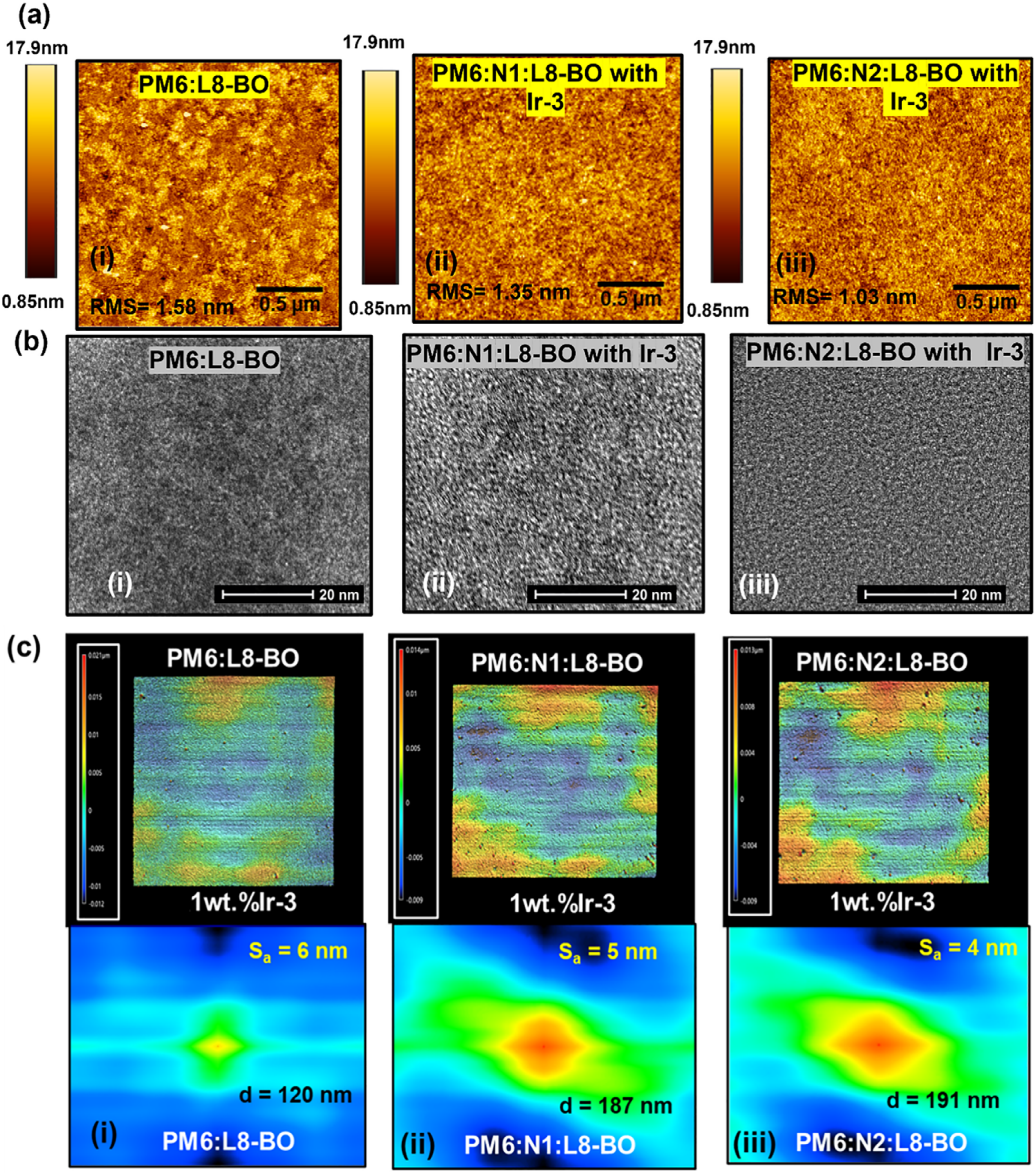
(a) AFM height images of (i) PM6:L8‐BO, (ii) PM6:N1:L8‐BO, and (iii) PM6:N2:L8‐BO doped with 1 wt.% Ir‐3. (b) TEM images of (i) PM6:L8‐BO, (ii) PM6:N1:L8‐BO, and (iii) PM6:N2:L8‐BO doped with 1 wt.% Ir‐3, showing individual fibril widths of 15–25 nm. (c) 3D laser confocal microscopy images illustrating the evolution of long‐range structural periodicity (d) and surface area roughness (S_a_) from the binary blend (d = 120 nm, S_a_ = 6 nm) to the N1‐based ternary blend (d = 187 nm, S_a_ = 5 nm), and to the N2‐based ternary blend (d = 191 nm, S_a_ = 4 nm).

### 2.8 Stability of PS‐OSCs

The operational and morphological stabilities of the PS‐OSCs were systematically evaluated under various conditions. Figure 8a shows the surface energies of the binary and ternary blends calculated from contact angle measurements (Figure S27) using the Owens‐Wendt method. The PM6:L8‐BO binary blend had a surface energy (γ) of 38.6 mJ m^−^^2^, while the N1 and N2‐based ternary blends containing 1 wt.% Ir‐3 showed progressively higher values of 41.0 and 42.8 mJ m^−^^2^, respectively. The higher γ of the N2‐based blend indicates better wettability and interfacial adhesion with charge transport layers, benefiting device stability and reproducibility [16]. The Flory‐Huggins interaction parameter (χ = 0.058), derived from these γ values, was lowest for the N2‐based blend, signifying better D/A miscibility and enhanced long‐term morphological stability (Figure S28). The thermal stabilities of unencapsulated devices annealed at 40–140°C in N2 atmosphere are shown in Figure 8b. The N2‐based ternary device retained 94% of its initial PCE after annealing at 140°C, significantly better than PM6:L8‐BO device, with 83% retention whereas, N1‐based ternary device has 88% retention (Figure S29). This enhanced thermal stability originates from the higher glass transition temperature (T_g_) of the N2 polymer donor (Figure S11) and the more ordered molecular packing observed by GIWAXS (Figure 6), which suppresses thermally induced phase separation and reduces recombination center formation [11]. The storage stability of encapsulated devices stored in an N_2_ glovebox at 25°C in the dark for 1000 h is shown in Figure 8c. The N2‐based champion device retained 89% of its initial PCE, outperforming the binary PM6:L8‐BO device (75% retention), depicting superior intrinsic stability under inert conditions (Figure S29). The superior storage stability of the N2‐based device is consistent with its balanced charge transport (µ_h_/µ_e_ = 1.03) and reduced trap‐state density, which minimizes charge accumulation and degradation [15]. The light‐soaking stability test (Figure 8d) was conducted under continuous AM 1.5G illumination at 100 mW cm^−^^2^ in a dry room environment (humidity: 30%–40%) over 3600 h. The N2‐based ternary device retained 53% of its initial PCE, notably outperforming the N1‐based device, with a 42% retention rate. This indicates superior photostability due to its face‐on orientation and effective π‐π suppression of bimolecular recombination, via balanced charge transport and reduced heat generation [12]. The complete stability data, including time‐dependent PCE, *V*_oc_, *J*_sc_, and FF evolutions, are provided in Figure S29. These results demonstrate that the combined synergy of the N2 polymer donor and Ir‐3 sensitizer enhances both initial device efficiency (PCE = 18.6%) and long‐term operational stability, making this system a promising candidate for practical photovoltaic applications [18, 19].

**FIGURE 8:**
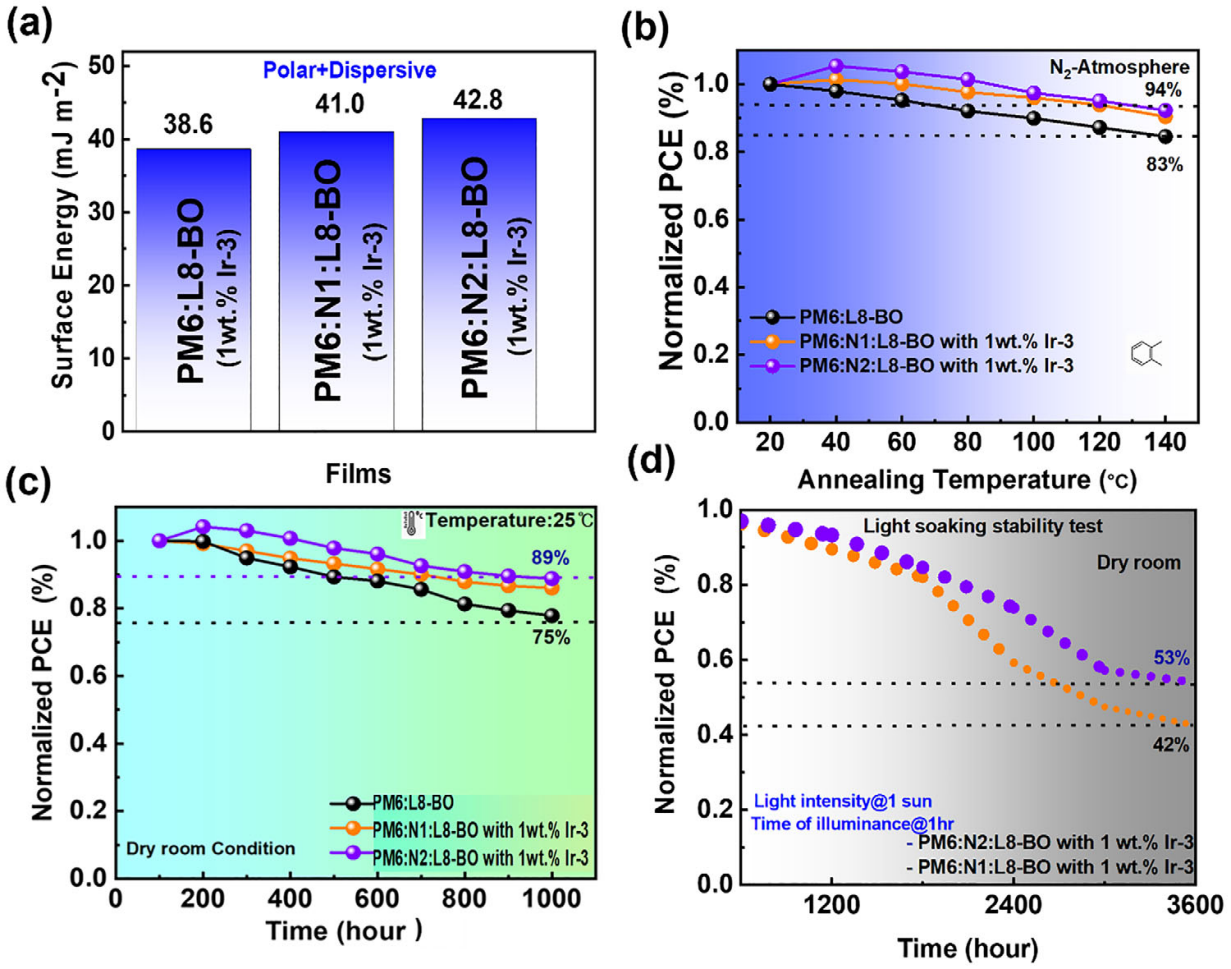
(a) Surface energies of the PS‐OSCs‐based binary and ternary devices with 1 wt.% Ir‐3. (b) Thermal stabilities of air‐processed N1 and N2 ternary blend devices. (c) Stabilities of ternary devices in a nitrogen environment inside a glove box. (d) Light‐soaking stability test of ternary PS‐OSC devices.

## 3 Conclusions

In summary, we designed a cooperative PSS for extending the performance limitations of ternary PS‐OSCs. The best device, based on the PM6:N2: L8‐BO doped with 1 wt.% Ir‐3, exhibits a remarkable PCE of 18.6%, with a *V_oc_* of 0.88 V, *J_sc_* of 27.6 mA cm^−^^2^, and FF of 76.7%. This efficiency is much higher than the binary host PCE of 16.0%. The enhanced efficiency is attributed to the dual benefit of host engineering and molecular sensitization. The N2 donor polymer forms an optimal morphology with a preferential face‐on orientation and balanced charge transport (µ_h_/µ_e_ = 1.03), while the Ir‐3 complex functions as a multifunctional additive, acting as a broad‐spectrum sensitizer via TTET, a *V_oc_* boosting mediator that suppresses non‐radiative recombination, and that mitigates trap‐assisted recombination and non‐radiative losses. Additionally, the best device demonstrates exceptional stability, retaining 89% of its initial PCE after 1000 h of inert N2 storage and showing greater photostability under continuous AM 1.5G light soaking over 3600 h with 53% retention as compared to N1‐based devices 42% retention. This work provides a universally applicable, environmentally friendly, and halogen‐free solvent framework, offering a broad and scalable strategy for the design of next‐generation PS‐OSCs.

## 4 Experimental Section

Supporting Information contains further information and characterization data.

## Author Contributions

P. Yadav and L. Guohong both contributed equally to this work. P. Yadav: conceptualization, device fabrication, DFT calculations, photovoltaic characterization, and writing the original draft. L. Guohong: synthesis and NMR analysis. H. Kim and S. Kim: spectral analysis. M. Kim: experiment, analysis, and measurement. Y. Ma: electrochemical and optical characterization. J. Lim: analysis and experiments. T. Jang: TAS characterization and analysis. Y. Y. Kim: GIWAXS measurements. R. Kumaresan is accountable for Ir‐3 complex synthesis and editing. J. Yoon did GIWAXS analysis and S. H. Jin was responsible for supervision, acquiring funding, administering the project, and reviewing.

## Conflicts of Interest

The authors declare no conflicts of interest.

## Supporting information




**Supporting File**: advs78199‐sup‐0001‐SuppMat.docx.

## Data Availability

The data that supports the findings of this study are available in the supplementary material of this article.
