# Supplementary material for: Phosphor‐Sensitized Ternary Organic Solar Cells Achieving 18.6% PCE via Ir(III)‐Mediated Triplet Energy Transfer and Non‐Radiative Recombination Suppression
Source: Adv Sci (Weinh). 2026 Oct 8:e78199. Online ahead of print. doi: 10.1002/advs.78199 (PMC13650016; doi:10.1002/advs.78199)
Supplement: Supplementary file 1 [file ADVS-9999-e78199-s001.docx]

Supplementary Information

**Phosphor-sensitized Ternary Organic Solar Cells achieving 18.6% PCE via Ir(III)-mediated Triplet Energy Transfer and Non-radiative Recombination Suppression**

*Priyanka Yadav^1^,* *Liu Guohong^1^, Hyerin Kim^1^, Songhyun Kim^1^, Minsung Kim^1^, Yeongbin Ma, Jisan Lim^1^, Young Yong Kim^2^,* *Taehyung Jang^3^, Raja Kumaresan^1*^, Jinhwan Yoon^4^*, and Sung-Ho Jin^1^**

P. Yadav, L. Guohong, H. Kim, S. Kim, M. Kim, Y. Ma, J. Lim, Y. Y. Kim, T. Jang, R. Kumaresan*, J. Yoon***, and S.-H. Jin***

^1^Department of Chemistry Education, Graduate Department of Chemical Materials, Institute for Plastic Information and Energy Materials, Sustainable Utilization of Photovoltaic Energy Research Center (ERC), Pusan National University, Busandaehakro 63-2, Busan 46241, Republic of Korea.

E-mail addresses: [kumaresan1224.r@gmail.com](mailto:kumaresan1224.r@gmail.com) (R. Kumaresan), shjin@pusan.ac.kr (S. H. Jin)

^2^Beamline Division, Pohang Accelerator Laboratory, Pohang University of Science and Technology, Pohang 37673, Republic of Korea.

^3^Energy Spectroscopy Laboratory, Department of Chemistry, Gwangju Institute of Science and Technology (GIST), 123, Cheomdangwagi-ro, Buk-gu, Gwangju, 61005, Republic of  Korea.

^4^Department of Chemical Engineering, Center for Innovative Chemical Processes, Institute of Engineering, University of Seoul, 163 Seoul Sirip-daero, Dongdaemun-gu, Seoul, 02504, Republic of Korea.

*E-mail addresses: jyoon@uos.ac.kr (J. Yoon)

*Priyanka Yadav and Liu Guohong contributed equally to this work.*

**Experimental section**

**Materials:**

PM6 and L8-BO were purchased from Solarmer Materials. BDT and DTBDT was synthesized according to the procedure mentioned below. Zinc acetate dihydrate (99.9%), 2-methoxyethanol (99.8%), ethanolamine (99.5%), the o-xylene solvent, and all other chemicals were purchased from Sigma Aldrich (Korea) without further purification.

**Synthesis**

The synthetic route of monomer benzo[1,2-b:4,5-b′]dithiophene (BDT), Dithieno[2,3-d:20,30-d0]-benzo[1,2-b:4,5-b0] dithiophene(DTBDT),and 2,5-bis(5-bromo-4-(2-ethylhexyl)thiophen-2-yl)pyrazine (Pyz) are shown in **Scheme S1**. The detailed synthetic procedure for monomer (a) was explained in the previous reports.^1^ 3-bromothiophene was reacted with 1,3-Bis(diphenylphosphino)propane]dichloronickel (II) and 2-ethyl hexyl magnesium bromide to obtain compound **2**. In the presence of TEMED compound **2** was treated with tributyltin chloride to yield compound **3**. The main pyrazine acceptor unit **4** was prepared via a Stille coupling reaction between 2,5-dibromopyrazine and compound **3**. Finally, the **Pyz** acceptor material was obtained by N-bromosuccinimide (NBS) bromination. The detailed synthetic procedures for monomer (b) (compound **10**) are explained in the previous reports.^2^ Further, the monomer (c) (compound **15**) was synthesized following the previous literature reports.^3^ The monomer structure was confirmed using ^1^H NMR and ^13^C NMR spectroscopy **(Figure S1).**

**Synthesis of 3-(2-ethylhexyl)thiophene (2):** 3-bromothiophene (2 g, 12.22 mmol) was added in 50 ml two-neck round bottom flask under a nitrogen atmosphere, thereafter 20 mL of anhydrous tetrahydrofuran was injected and stirred under N_2_ atmosphere for 10 min. [1,3-Bis(diphenylphosphino)propane]dichloronickel (II) (0.066 g, 0.12 mmol) was inserted quickly and purged until the two-neck round bottom flask was filled with N_2_ conditions. 2-ethyl hexyl magnesium bromide (3.2 g, 14.72 mmol) was added into the flask. The mixture was refluxed at 75ºC and stirred overnight in an oil bath. Water was used to quench the reaction, dichloromethane to extract it, and was dried over anhydrous sodium sulphate. Following the removal of the organic layer, the product was processed using column chromatography with hexane as an eluent, compound **1** as a colourless oil (1.95 g, Yield: 81%). ^1^H NMR (400 MHz, CDCl_3_) δ 7.26-7.24 (dd, 1H), 6.94-6.92 (dd, 1H), 2.59 (d, 2H), 1.73–1.59 (m, 1H), 1.34-1.21 (m, 8H), 0.91-0.84 (m, 6H).

**Synthesis of tributyl(4-(2-ethylhexyl)thiophen-2-yl)stannane (3):** In 50ml 2-neck round bottom flask, compound 1(1g, 5.09 mmol) was dissolved in 15ml anhydrous THF under N_2_ conditions. The TMEDA (1.143 mL, 7.64 mmol) was injected at -78ºC. After 10 minutes, the n-BuLi (2.5 M in hexanes, 3.259 mL, 8.15 mmol) was added to the flask dropwise and stirred for 1.5 hours. Tributyltin chloride (2.072 mL, 7.64 mmol) was added dropwise within 10 minutes to the flask and stirred for 2 h. Distilled water was used to dilute the mixture, which was subsequently separated with dichloromethane and dried over anhydrous sodium sulfate. The mixture was dried without any purification after evaporating the organic layer. Compound **2** as a yellow oil.

**Synthesis of 2,5-bis(4-(2-ethylhexyl) thiophen-2-yl) pyrazine (4):** Tributyl(4-(2-ethylhexyl) thiophen-2-yl) stannane (0.2 g, 0.84 mmol) was added in a 50 ml two-neck round bottom flask under a nitrogen atmosphere. 1,4-dibromopyrazine (2.45 g, 5.04 mmol) was added. Later, Tetrakis(triphenylphosphine)palladium (0.49 g, 0.04 mmol) was added under dark conditions and stirred while preventing light from entering. The reactants were heated for 24 hours at 110 °C, cooled, and poured into water to be extracted with dichloromethane. On top of anhydrous Na_2_SO_4_, the organic layer was dried. By using silica gel chromatography and dichloromethane (30% in hexane), the crude chemical was refined. The compound obtained was a yellow solid. (0.38 g, Yield: 97%). ^1^H NMR (400 MHz, CDCl_3_) δ 7.39 (s, 2H), 6.97 (a, 2H), 2.52 (d, 4H), 1.53 (s, 2H), 1.28-1.18 (m, 16H), 0.85-0.81 (m, 12H).

**Synthesis of 2,5-bis(5-bromo-4-(2-ethylhexyl)thiophen-2-yl)pyrazine (5) (monomer b):** In a 25 ml round bottom flask, 2,5-bis(4-(2-ethylhexyl)thiophen-2-yl)pyrazine(0.13 g, 0.277mmol) was dissolved in 10ml of THF within nitrogen conditions. NBS (0.12 g, 0.69 mmol) was quickly added under dark conditions, sealed well to prevent light from passing through, and stirred overnight. Water was used to quench the reaction, dichloromethane to extract it, and was dried over anhydrous sodium sulphate. Following the removal of the organic layer, the product was processed using column chromatography with hexane: dichloromethane (4:1) as an eluent. The compound obtained was a yellow solid. (0.62 g, Yield: 92%). ^1^H NMR (400 MHz, CDCl_3_) δ 8.66 (s, 2H), 7.23 (s, 2H), 2.47 (d, 4H), 1.59 (m, 2H), 1.29-1.24 (m, 16H), 0.86-0.82 (m, 12H) ^13^C NMR (500 MHz, CDCl_3_) δ 146.01, 143.61, 140.76, 139.40, 127.07, 124.29, 77.29, 77.04, 76.78, 40.32, 40.17, 34.60, 32.45, 29.72, 28.88, 25.61, 23.06, 14.16, 10.85.

**Synthesis of poly[2-(5-(4,8-bis(5-(6-((2-hexyldecyl)oxy)naphthalen-2-yl)thiophen-2-yl)-6-methylbenzo[1,2-b:4,5-b']dithiophen-2-yl)-4-(2-ethylhexyl)thiophen-2-yl)-5-(4-(2-ethylhexyl)-5-methylthiophen-2-yl)pyrazine] (NBDT-Pyz (N1)):** In a 10 mL microwave tube, Monomer (a) (0.32 mmol), Monomer (b) (0.32 mmol) and anhydrous chlorobenzene (1 mL) were stirred under nitrogen conditions. After 20 minutes, Pd_2_(dba)_3_ (10 mol %), and (*o*-tol)_3_P (20 mol %) were added. Under nitrogen conditions, the reactants were stirred for 15 minutes. After being inserted into the reactor, the microwave tube was heated for 1.5 hours to 135 °C. After reaching room temperature, the mixture was put into methanol to form a precipitate. Later, Soxhlet extraction was used for purification. Processed in the order of methanol, hexane, acetone, and chloroform. Evaporating chloroform yielded the polymer. (81%) (Scheme **2**).

**Synthesis of Ir-III complex**

**Synthesis of 2-chloro-N-(2-ethylhexyl)pyridin-3-amine (1)**. A solution of 3-amino-2-chloropyridine (10 g, 77.78 mmol) in anhydrous tetrahydrofuran (THF) (100 mL) was added to a suspension of sodium hydride (NaH) (3.1 g, 77.78 mmol, 60% dispersion in oil) and THF (50 mL) under N_2_ atmosphere. The reaction mixture was stirred at room temperature (RT) for 3 h. Afterwards, 2-ethylhexyl bromide (11.66 mL, 77.78 mmol) was added and the reaction mixture was heated to 40 ^o^C for 9 h. After cooling to RT, the solvent was removed and the crude material dissolved in DCM and filtered to remove any salt. The filtrate was purified by column chromatography on silica gel using ethyl acetate (EtOAc):n-hexane (10:90% v/v) as an eluent to afford compound **1** as a pale yellow liquid (8.5 g, yield: 85%). ^1^H NMR (300 MHz, CDCl_3_, δ): 7.64-7.63 (d, 1H), 7.06-7.02 (m, 1H), 6.84-6.81 (d, 1H), 4.33 (s, 1H), 3.01 (t, 2H), 1.57-1.55 (d, 1H), 1.41-1.28 (m, 8H), 0.87 (m, 6H).

**Synthesis of N-(2-chloropyridin-3-yl)-N-(2-ethylhexyl)benzamide (2).** NaH (1.66 g, 41.50 mmol) was added to anhydrous THF (30 mL) and the mixture was cooled to 0 ^o^C under N_2_ atmosphere. Compound **(1)** (5 g, 20.75 mmol) was added to the reaction mixture and stirred for 1 h. After that, 3-benzoyl chloride (4.25 mL, 31.15mmol) was added and stirred for 8 h at RT. After completion of the reaction, the reaction mixture was quenched by the addition of water (50 mL). Finally, the organic layer was extracted with DCM, washed with brine solution and dried over anhydrous Na_2_SO_4._ The solvent was evaporated off, and the solid residue was purified by column chromatography on silica gel using EtOAc:n-hexane (20:80% v/v) as an eluent to afford **6** as a white solid (3.6 g, yield: 72%). ^1^H NMR (300 MHz, CDCl_3,_ δ): 8.24 (s, 1H), 7.71-7.37 (d, 2H), 7.13-7.05 (d, 3H), 6.85-6.77 (d, 2H), 4.32 (s, 1H), 3.43-3.34 (t, 1H),1.46-1.25 (m, 8H), 0.89-0.82 (m, 6H).

**Synthesis of 5-(2-ethylhexyl)benzo[c][1,5]naphthyridin-6(5*H*)-one (3).** The mixture of compound **(6)** (1.25 g, 3.34 mmol), Pd(PPh_3_)_4_ (0.19 g, 0.16 mmol), Na_2_CO_3_ (1.76 g,16.70 mmol), and of DMA (30 mL) was stirred at 150 ^o^C for 24 h. Then the reaction mixture was cooled to RT and water (50 mL) was added. The resulting mixture was extracted with DCM (100 mL) and the organic phase was dried over anhydrous Na_2_SO_4_. The solvent was evaporated off, and the solid residue was purified by column chromatography on silica gel using EtOAc:n-hexane (10:90% v/v) as an eluent to afford **7** as a colorless liquid (0.8 g, yield: 70%). ^1^H NMR (300 MHz, CDCl_3,_ δ): 8.88-8.85 (d, 1H), 8.57-8.49 (t, 2H), 7.85-7.80 (t, 1H), 7.68-7.66 (d, 2H), 7.46-7.42 (m, 1H), 4.37-4.28 (d, 2H), 1.19 (d, 1H), 1.40-1.27 (m, 8H), 0.95-0.86 (m, 6H).

**Synthesis of iridium dimer complex (5):** Compound (**3**) (1.5 g, 53.91 mmol) and IrCl_3_·3H_2_O (0.76 g, 134.77 mmol) were added to a mixture of 2-ethoxyethanol and water (40 mL, 3:1 v/v). The reaction mixture was stirred at 120 ^o^C for 20 hrs and cooled to RT. The yellow precipitate was filtered, after that washed with water (60 mL) and methanol (10 mL), and subsequently dried under vacuum to afford dimer compound **5** as a yellow solid.

**Synthesis of bis[5-(2-ethylhexyl) benzo[c] [1,5] naphthyridin-6(5H)-one] (picolinate) iridium (III) (Ir3):** A solution of compound (**10**) (0.5 g, 0.29 mmol), 2-picolinic acid (0.18 g, 1.48 mmol) and Na_2_CO_3_ (0.30 g, 2.90 mmol) in 2-ethoxyethanol (25 mL) was stirred at 120 ^o^C under N_2_ for 12 h. After cooling to RT, the reaction mixture was poured into water and extracted with DCM. The organic layer was dried over anhydrous Na_2_SO_4_ and concentrated under vacuum and the obtained residue was purified by silica gel column chromatography using MeOH: EtOAc (40:60% v/v) as an eluent to afford **Ir-3** as a yellow solid (0.3 g, yield: 60%). ^1^H NMR (400 MHz, DMSO-d_6_, δ): 8.45-8.44 (d, 1H), 8.17-8.05 (m, 3H), 7.85-7.75 (m, 1H), 7.73-7.59 (m, 5H), 7.57-7.47 (m, 2H), 7.23-7.01 (m, 2H), 6.58-6.52 (m, 1H), 6.39-6.36 (dd, 1H), 4.42-4.25 (m, 4H), 2.03-1.87 (m, 2H), 1.35-1.16 (m, 18H), 0.91-0.70 (m, 12H); ^13^C NMR (75 MHz, CDCl_3_, δ): 174.4, 160.8, 160.0, 149.2, 148.4, 146.5, 145.5, 145.5, 143.9, 143.9, 139.2, 137.9, 131.9, 131.9, 129.4, 124.5,129.7,129.7, 129.4, 128.0,128.0, 127.3, 124.3, 124.3, 123.7, 123.7, 121.6, 121.6, 49.7, 49.7, 37.1, 37.1, 32.0, 32.0, 29.3, 29.3, 26.0, 26.0, 23.0, 23.0, 15.2, 14.1, 14.1. FAB^+^ MS (m/z): [M + H] ^+^ calcd. for C_46_H_50_IrN_5_O_4_, 929.14; found, 930.35.

**Fabrication of** **phosphor sensitized organic solar cells (PS-OSCs)**

The organic solar cells (OSCs) devices were fabricated with an inverted configuration of ITO/ZnO/active layer/MoO_3_/Ag. The Indium tin oxide (ITO)-coated glass with a sheet resistance of 10-15 ohm/square was cleaned by a surfactant scrub and then under an ultrasonic bath (20 min), beginning with deionized water followed by acetone and isopropyl alcohol. Then were treated by UV-ozone for 20 min. The ZnO layer was deposited by spin-coating under 3000 rpm for 30 sec on top of the ITO substrate annealed at 180 °C for about 20 min. Then substrates are kept in air atmosphere for few minutes and transferred to N_2_ glove box. The host PM6:L8-BO blend solutions were prepared using *o*- xylene solvent. The concentration of PM6 in the blend solution is 17 mg mL^-1^ (polymer donor: acceptor = 1:1.2 by weight) and were stirred for 8h before use. In the blends, the polymer donors were introduced in weight ratio of polymer are 0.8:0.2:1.2. The binary and ternary active layers were spin-coated on ZnO film with resulting thickness of 110 nm, and then annealed at 100 ℃ for 10 min. After that, 5nm MoO_3_ and 120 nm Ag were successively deposited on the active layer under high vacuum condition (5×10^-6^ Torr) and active area of 0.06 cm^2^.

**Measurements and characterization of the PS-OSCs**

**^1^H NMR and ^13^C NMR measurements:**

^1^H NMR and ^13^C NMR spectra at 300 MHz were recorded on a spectrometer (Varian Gemini-300).

**Gel permeation chromatography (GPC):**

The *M*_n_ and *M*_w_ of N1 and N2 were measured by (GPC) using PL gel 5 μm MLXED-C column on an Agilent 1100 series liquid chromatography system with tetrahydrofuran (THF) as an eluent, which was calibrated using polystyrene standards.

**Cyclic voltammetry (CV):**

CV measurements were conducted in an anhydrous chloroform (CHCl_3_) solvent by a CHI 600C potentiate (CH Instruments) at a scan rate of 100 mV/s with supporting electrolyte (0.1 M TBAClO_4_). A cell consists of 3 electrode Ag/AgCl. The reference electrode (calibrated by the ferrocene/ferrocenium redox couple, Fc/Fc^+^), platinum (Pt) as the working electrode, and Pt act as counter electrode and the polymerization was conducted in CEM-focused microwave TM synthesis system (CEM Discover-S).

**Thermogravimetric analysis (TGA) and different scanning calorimetry (DSC)**: were conducted on a Mettler Toledo TGA/SDTA 851e analyser under nitrogen atmosphere at a heating rate of 10 ^o^C/min.

**UV-Vis Absorbance:**

The absorption spectra of the pristine and blend states were measured on a UV-1800 spectrophotometer (Shimadzu Scientific Instruments) and Hitachi F-4500 fluorescence spectrophotometers at room temperature. The absorption coefficient (α) value was calculated using the following equation; (α) = 2.303*(A/l) (in cm), where A-absorbance and l-thickness (Energy Environ. Sci., 2017, 10, 258)

**PL Measurement:**

Photoluminescence (PL) measurements were carried out using a compact fluorescence lifetime spectrometer C11367 at room temperature.

**Density Functional Theory Study:**

Frontier molecular orbital distribution, energy levels for N1 and N2 polymers were obtained by DFT calculation with B3LYP and 6-31G* basis set. By simplifying the long side chains with methyl groups to understand the influence of alkoxy and Napthaloxy group substitution on the molecular geometries and energy level of the molecules.

**Active Layer Thickness Analysis:**

The thickness of active layers was measured via the KLA Tencor Alpha-step IQ surface profilometer.

**Current-Voltage Analysis:**

Power conversion efficiency was measured under AM (air mass) 1.5G illumination at 100 mW cm^-2^ using a AAA solar simulator (Oriel Sol3A Class AAA solar simulator, model 940443A) calibrated with a standard photovoltaic cell equipped with a KG5 filter and computer controlled Keithley 2400 source measure unit. PS-OSCs device PCEs were measured in dry room condition.

**EQE Analysis:**

The incident photon to current conversion efficiency spectrum was measured using Oriel ® IQE-200TM equipped with a 250 W quartz tungsten halogen lamp as the light source and a monochromator, an optical chopper, a lock-in amplifier, and a calibrated silicon 4 photodetector. Before to the use of the light, the spectral response and the light intensity were calibrated using a monosilicon detector.

**Jph-Veff Measurement:**

Here, Jph is like J_L_-J_D_, where J_L_ and J_D_ are the current density in the light and dark, respectively. V_eff_ is the difference between V_0_ and V_appl_,where V_appl_ is the applied bias voltage and V_0_ is the voltage when Jph = 0

**Trap-Assisted Recombination:**

The slope of the semi-log plot of Voc versus Plight was determined using the formula; Voc ∝ n(kT/q)ln(Plight), (where k is the Boltzmann constant, T is the temperature, and q is the elementary charge), and this slope can be used to investigate trap assisted recombination.

**Hole and electron-only devices and charge carrier mobility measurements:**

Hole-only and electron-only devices were fabricated to measure the hole and electron mobilities of active layers using the space charge limited current (SCLC) method with hole-only device of ITO/PEDOT:PSS/activelayer/MoO3/Ag and electron-only device of ITO/ZnO/active layer/PDINO/Ag. The SCLC mobilities were calculated by MOTT-Gurney equation, which is described by: J= 9/8 ε_0_εr_μ_V^2^ /d ^3^ , where J is the current, E is the effective electric field, ε_0_ is the permittivity of free space (8.85×10^-12^ F m^-1^ ), ε_r_ is the material relative permittivity, d is the thickness of the active layer, μ is the hole or electron mobility and V is the effective voltage. The effective voltage can be obtained by subtracting the built-in voltage (V_bi_) and the voltage drop (V_s_) from the substrate’s series resistance from the applied voltage (V_appl_), V = V_appl_– V_bi_ – V_s_. The mobility can be calculated from the slope of the J 1/2 ~V curves.

**Trap-State Density Measurement:**

The trap-state density was determined according to the formula; Nt = (2ε_r_ε_0_VTFL)/(qL^2^ ), where, Nt is the trap-state density, VTFL is the trap-filling limit voltage, ε_r_ is the relative dielectric constant (3), ε_0_ is the vacuum permittivity (8.85×10-14 CV-1 cm-1 ), L is the thickness of active layer (110 nm), and q is the elementary charge (1.6×10-19 C).

**Morphological Analysis:**

The surface morphology of the blend films was characterized by AFM images which were acquired with a XE-100 (park system corp) in tapping mode. Samples were prepared under optimized conditions and the measurement was carried out in tapping mode. For bulk morphology analysis, TEM images were acquired with a H-7600 (HITACHI)/electron microscopy

**Impedance Study:**

The impedance response was measured over the range of 1 Hz to 1 MHz with oscillation amplitude of 15 mV under dark condition (Bio-Logic VMP-3). The experimental data were simulated using commercial Z-view software to estimate the values for each component of the corresponding equivalent circuits.

**Contact Angles Study:**

The contact angles were measured using a contact angle 101 measuring system (Plasma systems and materials, South Korea).

**Flory–Huggins interaction parameter:**

The Flory-Huggins interaction parameter was also calculated using the following equation with the surface energy values of the donor and acceptor component (γdonor and γacceptor, respectively): χ∝[(γdonor) ^2^ -(γacceptor) ^2^ ] ^2^ , χ = Flory–Huggins interaction parameter and γ = Surface energy.

**Stability Analysis:**

The PS-OSCs devices were tested for (thermal stability) in a dry room environment condition. The devices at various annealing temperatures between 40 to 140 °C in both the open air (relative humidity: 30~40%) and the nitrogen atmosphere. Moreover, thermal stability was measured in a dry environment at 25 °C stability of PS-OSCs.

**Scheme S1.** Synthetic route of Monomers.

**Scheme S2.** Synthetic route of Ir-3 complex.

**Figure. S1.** ^1^H NMR of compound 5.

**Figure. S2.** ^13^C NMR of Compound 5.

**Figure. S3.** ^1^H NMR of Ir-3 complex.

**Figure. S4.** ^13^C NMR of Ir-3 complex.

**Figure. S5.** Mass spectra of Ir-3 complex.

**Figure. S6.** Cyclovoltametric analysis of N1 and N2 polymers

**Figure. S7.** Cyclovoltametric analysis of Ir-3.

**Figure. S8.** Temperature-dependent UV-visible absorption spectra showing A (0-1) /A (0-0) vibronic transitions of N1.

**Figure. S9.** Temperature-dependent UV-visible absorption spectra showing A (0-1) /A (0-0) vibronic transitions of N2.

**Figure. S10.** Differential scanning calorimetry (DSC) curves polymers (N1 and N2).

**Figure. S11.** Differential scanning calorimetry (DSC) curves of Ir-3.

**Figure. S12.** Normalized absorption spectra of binary and ternary blends of PM6:L8-BO, PM6:N1: L8-BO and PM6:N2: L8-BO doped with 1 wt. % Ir-3.

**Figure. S13.** Steady state PL- quenching spectra of PM6:N1: L8-BO and PM6:N2: L8-BO blend films without 1 wt. % Ir-3.

**Figure. S14**. DFT of (a) Top view (b) side view of N1 polymer.

**Figure. S15**. DFT of (a) Top view (b) side view of N2 polymer.

**Figure. S16.** Binary optimized *J-V* graph of N1and N2 based PS-OSCs.

**Figure. S17.** Ternary optimized (a) *J-V* and (b) EQE graphs of N1and N2 based PS-OSCs without Ir-3 dopant.

**Figure. S18.** Thickness optimized *J-V* graph of N2 based ternary PS-OSCs with 1 wt. % of Ir-3 doped.

**Figure. S19.** Blend ratio optimized *J-V* graph of N2 based on ternary PS-OSCs with 1 wt. % of Ir-3 doped.

**Figure. S20.** Solvent optimized *J-V* graph of N2 based on ternary PS-OSCs with 1 wt. % of Ir-3 doped .

**Figure. S21**. TRPL decay curves of the Ir-3-free PM6:N1:L8-BO and PM6:N2:L8-BO ternary blend films, fit with a bi-exponential model.

**Figure. S22.** Dark current densities of optimized Ir-3 doped (a) N1 and (b) N2 based ternary PS-OSCs.

**Figure. S23.** Trap-state densities of optimized N1 based ternary PS-OSCs with 1 wt. % of Ir-3 doped.

**Figure. S24.** Trap-state densities of optimized N2 based ternary PS-OSCs with 1 wt. % of Ir-3 doped.

**Figure. S25.** Impedances of 1 wt. % of Ir-3 doped binary and ternary PS-OSCs.

**Figure. S26.** 2D Giwax images of 1 wt. % Ir-3 doped N2-based ternary film.

**Figure. S27.** Contact angles of 1 wt. % Ir-3 doped binary and ternary PS-OSCs in H_2_O and DIM.

**Figure. S28.** Flory-Huggins vs PCE of binary and ternary PS-OSCs with1 wt. % Ir-3.

**Figure. S29**. Nitrogen atmosphere storage stability in glove box of PS-OSCs (a) PM6:L8-BO (b) PM6:L8-BO with Ir-3 (c) PM6:N1: L8-BO with Ir-3 and (d) PM6:N2: L8-BO with 1 wt. % Ir-3 .

**Table S1.** The molecular weight of the Ir-3 complex and the two synthesized polymers.

| Polymer | M_n_ [g mol^-1^] | M_w_ [g mol^-1^] | PDI |
| --- | --- | --- | --- |
| Ir-3  PBDT-Pyz (**N1**)  PBDTT-Pyz (**N2**) | -  60954  108120 | 930.35  200000  283450 | -  3.28  2.62 |

**Table S2.** Solubility test of the three guest polymers in different green solvents using Ir-3, N1 and N2 with 20 mg/ml concentration study.

| Polymer | CB | Toluene | o-xylene | CF |
| --- | --- | --- | --- | --- |
| Ir-3  PBDT-Pyz **(N1)**  PBDTT-Pyz **(N2)** | **Δ**  **Δ**  **Δ** | **Δ**  **◊**  **Δ** | £  £  £ | **Δ**  **Δ**  **Δ** |

Where, **◊**: moderate, **Δ**: good, and: £ excellent.

**Table S3.** Detailed optical, thermal, and electrochemical properties of N1 and N2 polymer donors.

| Polymer | λ_max_^a)^ | λ_max_^b)^ | ^c)^  [M^-1^ cm^-1^] | E_g_^optd)^  [eV] | T_d_^e)^  [ºC] | HOMO^f)^ | LUMO^g)^ |
| --- | --- | --- | --- | --- | --- | --- | --- |
| N1 | 540 | 542 | 1.37 x 10^5^ | 1.96 | 376 | -5.59 | -3.63 |
| N2 | 544 | 544 | 1.50 x 10^5^ | 1.98 | 379 | -5.58 | -3.60 |

a) Measured in CHCl_3_ at a concentration of 10^-5^ mol L^-1^. ^b)^ Measured in neat films. ^c)^Absorption coefficient at λ_max_ in solution. ^d)^Optical bandgap (*E*_g_^opt^) calculated from the onset of UV-vis-spectrum, *E*_g_^opt^ =1240/λ. ^e)^ Temperature corresponding to 5% mass loss was determined by TGA at a heating rate of 10 ºC min^-1^ under N_2_. ^f)^ HOMO values were determined when CV measurements commenced. ^g)^ LUMO values were calculated from HOMO values using LUMO = HOMO + *E*_g_^opt^.

**Table S4.** Detailed Photophysical, thermal, and electrochemical properties of the Ir-3 complex.

| Ir (III) complex | T_g_/T_d_  (°C) | λ_PL_  (nm)^a)^ | | λ_PL_  (nm)^b)^ | τ  (μs) ^a)^ | HOMO  (eV)^c)^ | LUMO  (eV)^c)^ | Eg  (eV) | E_T_  (eV)^d)^ |
| --- | --- | --- | --- | --- | --- | --- | --- | --- | --- |
|  |  | CH_2_Cl_2_ | film | 77 K | CH_2_Cl_2_ |  |  |  |  |
| Ir-3 | 160**/**362 | 517 | 528 | 513 | 0.30 | -5.5 | -3.0 | 2.5 | 2.46 |

^a)^ Measured in degassed CH_2_Cl_2_ solution at RT (1×10^-5^ M). ^b)^ Measured in 2-MeTHF solution at 77 K. ^c)^ HOMO calculated from onset oxidation potential from CV, LUMO = E_g_ (optical) + HOMO. ^d)^E_T_ determined from low temperature phosphorescent spectra.

**Table S5.** Summary of PL quenching for ternary PS-OSCs with 1wt.% Ir-3.

| Active Layer | PL quenching effect (ΔPL_max_) | PCE (%) |
| --- | --- | --- |
| PM6: N1:L8-BO with 1wt.% Ir-3 | 94.03 % | 17.7 |
| PM6: N2:L8-BO with 1wt.% Ir-3 | 98.86% | 18.6 |

**Table S6.** Dihedral angles of pyrazine based-conjugated polymers donors and Ir-3 complex.

| Polymers | Q1  [degree]^a)^ | Q2  [degree]^a)^ | Q3  [degree]^a)^ | Q4  [degree]^a)^ | Q5  [degree]^a)^ | Q6  [degree]^a)^ |
| --- | --- | --- | --- | --- | --- | --- |
| PBDT-Pyz **(N1)** | 122.6 | 120.5 | 121.5 | 120.7 | 118.3 | 120.1 |
| PBDTT-Pyz **(N2)** | 122.6 | 120.4 | 121.5 | 120.7 | 118.3 | 121.0 |
| **Ir-3** | 78.98 | 97.23 | 91.03 | 80.82 | 93.28 | - |

^a)^ Calculated by DFT simulation**.**

**Table S7.** Summary of space charge limited current (SCLC) mobilities of binary guest polymer donor based small PS-OSCs with 1wt. % Ir-3.

| Active Layer | µ_h_  (cm^2^ V^-1^ s^-1^) | µ_e_  (cm^2^ V^-1^ s^-1^) | µ_h_: µ_e_ |
| --- | --- | --- | --- |
| PM6:L8-BO with 1wt.% Ir-3 | 5.03 × 10^-4^ | 4.49 × 10^-4^ | 1.12 |
| PM6:N1:L8-BO with 1wt.% Ir-3 | 6.52× 10^-4^ | 6.15 × 10^-4^ | 1.06 |
| PM6:N2: L8-BO with 1wt.% Ir-3 | 6.45 × 10^-4^ | 6.26 × 10^-4^ | 1.03 |

**Table S8.** The PS**-**OSCs with 1wt. % Ir-3 relative parameter obtained from *J_ph_-V_eff_* curves.

| Active layers | *J_ph_*  (mA/cm2 ) | *J_sat_*  (mA/cm2 ) | *G*_max_  (m^-3^ s^-1^) (×10^28^) | P (E, T)^a)^  (%) |
| --- | --- | --- | --- | --- |
| PM6:L8-BO with 1wt.% Ir-3 | 24.0 | 25.9 | 1.41 | 92.7 |
| PM6:N1:L8-BO with 1wt.% Ir-3 | 24.3 | 26.2 | 1.42 | 92.7 |
| PM6:N2: L8-BO with 1wt.% Ir-3 | 25.7 | 26.4 | 1.43 | 97.3 |

^a)^Under short-circuit condition, V*_appl_* = 0

Table S9. Photovoltaic parameters for binary guest polymer donor-based PS-OSCs with 1wt.% Ir-3 processed in o-xylene solvent under simulated solar illumination (AM 1.5G,100 mW/cm^2^).

| Active layer | *V_oc_*  (V) | *J_sc_*  (mAcm^-2^) | FF  (%) | PCE  (%) |
| --- | --- | --- | --- | --- |
| N1:L8-BO with 1wt.% Ir-3 | 0.78 | 15.6 | 67.5 | 8.21 |
| N2: L8-BO with 1wt.% Ir-3 | 0.79 | 16.5 | 69.0 | 8.99 |

**Table S10.** Photovoltaic parameters for ternary guest polymer donors (N1 and N2) based PS**-**OSCs without 1wt.% of Ir-3 doping processed in o-xylene solvent under simulated solar illumination (AM 1.5G,100 mW/cm^2^).

| Active layer | *V_oc_*  (V) | *J_sc_*  (mAcm^-2^) | *Integrated J_sc_*  (mAcm^-2^) | FF  (%) | PCE  (%) |
| --- | --- | --- | --- | --- | --- |
| PM6:N1:L8-BO | 0.86 | 26.8 | 25.6 | 73.8 | 17.0 |
| PM6:N2: L8-BO | 0.87 | 27.2 | 26.3 | 74.1 | 17.5 |

**Table S11.** Photovoltaic parameters for PS**-**OSCs based on PM6:N2:L8-BO(0.8:0.2:1.2) along with 1wt.% Ir-3 dopant with various thickness under simulated solar illumination (AM 1.5G,100 mW/cm^2^).

| Thickness  (nm) | *V_oc_*  (V) | *J_sc_*  (mAcm^-2^) | FF  (%) | PCE  (%) |
| --- | --- | --- | --- | --- |
| 90 ±5 | 0.85 | 25.1 | 65.8 | 14.0 |
| 100 ±10 | 0.86 | 26.1 | 76.3 | 17.1 |
| 110 ±15 | 0.88 | 27.6 | 76.7 | 18.6 |
| 120 ±20 | 0.87 | 26.6 | 75.5 | 17.4 |
| 125 ± 25 | 0.86 | 22.3 | 61.5 | 11.7 |

**Table S12.** Photovoltaic parameters for PS-OSCs based on PM6:N2:L8-BO with 1wt.% Ir-3 dopant under various blending ratio, processed in o-xylene solvent under simulated solar illumination (AM 1.5G,100 mW/cm^2^).

| Blend ratio | *V_oc_*  (V) | *J_sc_*  (mAcm^-2^) | FF  (%) | PCE  (%) |
| --- | --- | --- | --- | --- |
| 0:1:1.2 | 0.83 | 15.9 | 41.3 | 5.4 |
| 1:0:1.2 | 0.85 | 27.4 | 74.6 | 17.3 |
| 0.9:0.1:1.2 | 0.86 | 26.4 | 75.8 | 17.2 |
| 0.8:0.2:1.2 | 0.88 | 27.6 | 76.7 | 18.6 |
| 0.7:0.3:1.2 | 0.85 | 24.8 | 72.4 | 15.2 |

**Table S13.** Photovoltaic parameters for PM6:N2:L8-BO with 1wt.% Ir-3 dopant devices using halogenated and halogen-free solvents under simulated solar illumination (AM 1.5G,100 mW/cm^2^ ).

| Solvent | *V_oc_*  (V) | *J_sc_*  (mAcm^-2^) | FF  (%) | PCE  (%) |
| --- | --- | --- | --- | --- |
| Toluene | 0.86 | 24.2 | 68.5 | 14.2 |
| CB | 0.87 | 27.4 | 75.9 | 18.0 |
| CF | 0.86 | 26.2 | 74.6 | 16.8 |
| *o*-Xylene | 0.88 | 27.6 | 76.7 | 18.6 |
| CB +Toluene | 0.86 | 25.3 | 74.8 | 16.2 |

**Table S14.** Bi-exponential and tri-exponential TRPL decay fit parameters for the Ir-3-free and 1 wt.% Ir-3-doped PM6:N1:L8-BO and PM6:N2:L8-BO ternary blend films.

| Film Sample | Fitting  model | f1 (%) / τ1  (ns) | f2 (%) / τ2  (ns) | f3 (%) / τ3  (ns) | τ^a)^_avg_  (ns) | Adj. R² |
| --- | --- | --- | --- | --- | --- | --- |
| PM6:N1:L8-BO | Bi-exponential | 79.09 / 0.19 | 20.91 / 1.73 | - | 1.27 | >0.998 |
| PM6:N2:L8-BO | Bi-exponential | 80.01 / 0.16 | 19.99 / 1.52 | - | 1.12 | >0.998 |
| PM6:N1:L8-BO  (1 wt.% Ir-3) | Tri-exponential | 80.04 / 0.11 | 16.62 / 0.53 | 3.34 / 2.23 | 0.88 | >0.998 |
| PM6:N2:L8-BO  (1 wt.% Ir-3) | Tri-exponential | 83.80 / 0.10 | 13.87 / 0.43 | 2.33 / 1.78 | 0.58 | >0.998 |

^ᵃ)^ τ_avg_ is the intensity-weighted average lifetime, τ_avg_ = ΣAiτi² / ΣAiτi, computed from the origin fit values (Ai, τi) before rounding to the figures shown above. fi is the fractional amplitude contribution of each component, fi = Ai /ΣAj × 100%. Adjusted R² > 0.998 for all fits.
